# Putaminal hypermetabolism identifies Lewy body co‐pathology in Alzheimer's disease

**DOI:** 10.1002/alz.70920

**Published:** 2025-11-22

**Authors:** Sungwoo Kang, Seun Jeon, Yeoju Kim, Su‐Hee Jeon, Minsun Choi, Young‐gun Lee, Byoung Seok Ye, for the Alzheimer's Disease Neuroimaging Initiative

**Affiliations:** ^1^ Department of Neurology Hanyang Seoul Hospital Hanyang University College of Medicine Seoul Republic of Korea; ^2^ Metabolism‐Dementia Research Institute Yonsei University College of Medicine Seoul Republic of Korea; ^3^ Department of Neurology Ilsan Paik Hospital Inje University College of Medicine Goyang Republic of Korea; ^4^ Department of Neurology Yonsei University College of Medicine Seoul Republic of Korea

**Keywords:** Alzheimer's disease, Lewy body disease, metabolic imaging, seeding amplification assays

## Abstract

**INTRODUCTION:**

The clinical implications of brain hypermetabolism remain unexplored in Lewy body disease (LBD) co‐pathology in Alzheimer's disease (AD).

**METHODS:**

We investigated cognition, ^18^F‐fluorodeoxyglucose positron emission tomography (PET), and cerebrospinal fluid tau phosphorylated at threonine 181 (pTau_181_)/Aβ_42_ plus α‐synuclein seeding amplification assays (SAA) in controls, 217 SAA‐negative AD (AD^SAA−^), and 124 SAA‐positive AD (AD^SAA+^). Brain metabolism was assessed using subject residual profile (SRP) and standardized uptake value ratio (SUVR).

**RESULTS:**

Compared to AD^SAA−^, AD^SAA+^ showed putamen SRP hypermetabolism and middle occipital gyrus (MOG) SUVR hypometabolism. SAA positivity correlated with putamen SRP hypermetabolism independently of pTau_181_/amyloid beta 42 (Aβ_42_). Its interaction with pTau_181_/Aβ_42_ influenced MOG SUVR, showing increased MOG SUVR with higher pTau_181_/Aβ_42_ in AD^SAA+^. Putamen SRP hypermetabolism predicted faster cognitive decline and greater variability in both groups. MOG SUVR hypometabolism correlated with them only in AD^SAA−^. Adding putamen SRP hypermetabolism to models, including SAA positivity and AD signature hypometabolism, improved the prediction of cognitive decline/variability, whereas MOG SUVR did not.

**DISCUSSION:**

Putaminal hypermetabolism may serve as a robust metabolic marker of LBD co‐pathology in AD.

**Highlights:**

- LB co‐pathology in AD alters regional brain metabolism.
- SRP analyses capture putaminal hypermetabolism for SAA positivity.
- SUVR analyses emphasize occipital hypometabolism for SAA positivity.
- Occipital metabolism correlates positively with AD severity in mixed AD‐LB.
- Putaminal, not occipital, metabolism predicts cognitive change over AD‐related metabolism and SAA.

## 1 BACKGROUND

Alzheimer's disease (AD) is the most common cause of neurodegenerative disease, with approximately 43% to 60% of individuals with AD exhibiting concurrent Lewy body (LB) pathology.^1^, ^2^, ^3^ LB disease (LBD) is the second most common neurodegenerative disorder, characterized by the intraneuronal aggregation of misfolded α‐synuclein, and about 50% of LBD cases also exhibit AD pathology.^4^ Although previous *post mortem* studies consistently showed that mixed AD–LBD led to faster cognitive decline compared to pure disease,^4^, ^5^, ^6^, ^7^ the lack of in vivo biomarkers for LB pathology has hindered the clinical identification of mixed AD–LBD. However, the advent of α‐synuclein seeding amplification assays (SAA) has enabled the in vivo detection of LB pathology with high diagnostic accuracy.^8^, ^9^ Recent SAA‐based studies have confirmed that individuals with in vivo‐detected AD–LBD exhibit faster cognitive worsening,^10^, ^11^ reinforcing the clinical significance of detecting mixed disease, which may contribute to the optimization of disease‐modifying therapies.

Recent studies^10^, ^12^, ^13^ using ^18^F‐fluorodeoxyglucose (FDG)‐positron emission tomography (PET) and SAA data from the Alzheimer's Disease Neuroimaging Initiative (ADNI) database have consistently demonstrated an association between SAA positivity and occipital hypometabolism. Although LBD is typically associated with posterior‐occipital hypometabolism,^14^ putaminal hypermetabolism has also been consistently observed in dementia with Lewy bodies (DLB)^15^ and Parkinson's disease (PD).^16^ Pathological studies further indicate that LB pathology is associated with hypermetabolism in the bilateral putamen and anterior cingulate cortex, independent of AD pathology.^17^ Additionally, putaminal hypermetabolism has been linked to the severity of striatal dopaminergic degeneration in DLB.^18^, ^19^ Even in prodromal LBD, such as isolated rapid eye movement sleep behavior disorder, pallidal hypermetabolism, a key component of the basal ganglia along with the putamen, has been associated with striatal dopamine loss.^20^ Considering hypermetabolism in the basal ganglia in DLB without significant dopamine deficiency,^21^ metabolic alterations in putamen could serve as a potential biomarker for detecting LB pathology in AD.

In this study, we investigated the effect of SAA positivity on relative hypermetabolism and hypometabolism in cognitively impaired patients with AD, ranging from mild cognitive impairment (MCI) to dementia. We hypothesized that the putamen and occipital cortex would exhibit the greatest metabolic differences between pure AD and mixed AD–LBD. Based on our previous autopsy‐imaging correlation study, which demonstrated an interaction between AD and LB pathologies on occipital metabolism,^17^ we further hypothesized that putaminal relative hypermetabolism would serve as a more reliable biomarker for LBD than occipital hypometabolism among individuals with AD.

## 2 METHODS

### 2.1 Participants

We included 503 participants from the ADNI database who had magnetic resonance imaging (MRI), FDG imaging, and cerebrospinal fluid (CSF) biomarkers for both AD (described below) and α‐synuclein status. Participants were selected through a two‐step process. First, we identified individuals with complete MRI, FDG‐PET, and CSF SAA data, excluding those with poor imaging quality (*N* = 54), indetermined SAA results (*N* = 7), or MRI‐FDG scan intervals exceeding 2 years (*N* = 3). Second, we refined the cohort to include cognitively impaired participants with AD (i.e., MCI or dementia) and cognitively unimpaired neurologically normal controls (NC) with negative CSF biomarkers for both AD and α‐synuclein. We excluded NC individuals with a positive CSF AD biomarker, regardless of their SAA results (*N* = 42). Additionally, we excluded cognitively impaired individuals who were negative for CSF AD biomarker but positive for SAA (*N* = 56), as well as those who were cognitively impaired but had negative CSF biomarkers for both AD and α‐synuclein (*N* = 204). The final cohort consisted of 108 NC, 194 MCI, and 201 dementia participants. Details are provided in the flowchart (Figure S1). The inclusion and exclusion criteria, protocols for lumbar puncture, and CSF biomarker tests are described on the ADNI website (https://adni.loni.usc.edu/help‐faqs/adni‐documentation/). Information on *apolipoprotein ε E4* (*APOE4*) status was collected from the Biospecimen Results section of the ADNI website (https://ida.loni.usc.edu/explore/jsp/search/search.jsp?project=ADNI#studyFiles). Information on tremor and gait disturbance was collected from the Physical/Neurological Exams section of the Medical History records in the ADNI website and was used to determine whether these symptoms were present at any time point.

**RESEARCH IN CONTEXT**

1. **Systematic review**: The authors conducted a literature review through PubMed on brain metabolism, LB co‐pathology, and SAA in AD. Prior studies consistently reported that LB co‐pathology was associated with occipital hypometabolism assessed using SUVR. However, regional hypermetabolism, assessed using SRP, has not been explored in the context of AD.
2. **Interpretation**: This study demonstrated that SAA‐positive AD patients exhibited occipital hypometabolism when assessed by SUVR and putaminal hypermetabolism when assessed by SRP. Putaminal hypermetabolism was associated with faster cognitive decline and greater cognitive variability. Notably, in SAA‐positive AD patients, occipital metabolism was positively associated with AD biomarkers. Furthermore, only putaminal hypermetabolism, rather than occipital hypometabolism, enhanced the prediction of cognitive decline and variability beyond SAA positivity and AD signature metabolism. These findings suggest that SRP‐based putaminal hypermetabolism may serve as a more consistent marker of LB co‐pathology in AD than SUVR‐based occipital hypometabolism.
3. **Future directions**: Our findings underscore the potential of SRP‐based metabolic imaging in identifying LB co‐pathology in vivo among individuals with AD. Future studies should investigate the utility of putaminal hypermetabolism as a diagnostic or prognostic biomarker for AD–LBD spectrum disorders and explore its role in guiding personalized therapeutic strategies, especially in mixed‐pathology cases.

### 2.2 Neuropsychological assessments

Composite cognitive scores across several domains were previously validated in the ADNI cohort.^22^, ^23^ The memory composite was modeled based on components from the Rey Auditory Verbal Learning Test, the Alzheimer's Disease Assessment Scale–Cognitive Subscale (ADAS–Cog), and the Mini‐Mental Status Examination (MMSE). The language composite was modeled using animal and vegetable category fluency, the Boston naming test, MMSE language elements, following commands/object naming/ideational practice from ADAS–Cog, and Montreal Cognitive Assessment language elements, including letter fluency, naming, and repeating tasks. The visuospatial composite was modeled based on five dichotomous clock‐copying tasks (circle, symmetry, numbers, hands, and time) from the neuropsychological battery, the ADAS–Cog Constructional Praxis task, and the MMSE copy design task. The executive function composite was modeled using animal and vegetable category fluency, Trail Making Test Parts A and B, digit span backward, digit symbol substitution from the revised Wechsler Adult Intelligence Scale, and the circle, symbol, numbers, hands, and time items from a clock‐drawing task. Further details on these composite measures can be obtained on the ADNI website (https://adni.bitbucket.io/reference/docs/UWNPSYCHSUM/adni_uwnpsychsum_doc_20200326.pdf). MMSE and ADAS–Cog scores were used as measures of general cognition. Annual cognitive changes were calculated by taking the difference between baseline and final scores for general cognition (MMSE, ADAS–cog) and domain‐specific scores (memory, language, visuospatial, and executive) and dividing it by the follow‐up duration. Cognitive variability was assessed as the standard deviation of multiple measurements over time.

### 2.3 CSF biomarkers in the ADNI database

The levels of CSF Aβ_42_ and tau phosphorylated at threonine 181 (pTau_181_), previously measured using the Roche Elecsys Immunoassay at the ADNI Biomarker Core Laboratory of the University of Pennsylvania Medical Center,^24^ were downloaded from the ADNI database on September 9, 2024. To represent Alzheimer's pathology, we calculated the pTau_181_/Aβ_42_ ratio. Since the literature reports a cutoff value of 39.20 for the pTau_181_/Aβ_42_ ratio in relation to amyloid PET from the ADNI database,^24^ we considered the reciprocal, 0.0255, as the threshold for AD biomarker positivity.

The α‐synuclein status was determined using the Amprion SAA^25^ and was obtained within 2 years of FDG‐PET imaging (based on the ADNI database on September 9, 2024). The mean interval was 18.2 ± 45.4 days in the NC group, 33.5 ± 137.0 days in the SAA‐negative AD (AD^SAA−^) group, and 31.5 ± 140.0 days in the SAA‐positive AD (AD^SAA+^) group.

### 2.4 Amyloid PET Centiloid values in the ADNI database

Individual amyloid burden quantified in Centiloid (CL) units was downloaded from the ADNI Image & Data Archive (accessed June 2025). For each participant, we selected the amyloid‐PET scan closest to the FDG‐PET acquisition within a 1‐year interval (mean ± SD = 10.5 ± 18.4 days). CL values were derived by (i) averaging uptake across the frontal, anterior/posterior cingulate, lateral parietal, and lateral temporal cortices; (ii) normalizing this value to whole‐cerebellum standardized uptake value (SUV); and (iii) converting the resulting SUV ratio (SUVR) to CL units using the validated linear transformations for each amyloid tracer. Detailed CL protocols are available at https://adni.loni.usc.edu/help‐faqs/adni‐documentation.

### 2.5 Image preprocessing for FDG‐PET

Based on the SAA test date, the FDG‐PET scan closest in time was selected for each individual, and contemporaneous T1‐weighted MRI images were included in the imaging analyses. Both FDG‐PET and T1‐weighted MRI images were downloaded from the ADNI database (https://adni.loni.usc.edu/). Detailed information on the MRI and PET scans, including acquisition protocols, is available elsewhere (https://adni.loni.usc.edu/data‐samples/adni‐data/neuroimaging/). Image processing was conducted using the FMRIB Software Library (FSL, http://www.fmrib.ox.ac.uk/fsl/) and Advanced Normalization Tools (ANTs). T1‐weighted images were corrected for intensity inhomogeneity, skull‐stripped, and aligned to the ADNI‐Montreal Neurological Institute (MNI) atlas, serving as a T1‐weighted template for older adults.^26^, ^27^ Using a hidden‐Markov random field model with an expectation–maximization algorithm, brain tissues were classified into white matter, gray matter (GM), and CSF.^28^ The generated GM probability map was then non‐linearly registered to the ADNI‐MNI template. Striatal regions were identified using the FMRIB Integrated Registration and Segmentation Tool (FIRST) algorithm and included within the GM tissue class.^29^ GM probability maps were then averaged across individuals, binarized using a 30% threshold, and used to generate a GM mask for further statistical analyses.

Each subject's FDG‐PET image was linearly co‐registered to their T1‐weighted MRI using a rigid body transformation. SUVR maps were generated using the cerebellar cortex as a reference region. These SUVR maps were spatially normalized to the ADNI‐MNI template using non‐linear warping fields derived from the T1‐weighted MRI processing and subsequently smoothed with a 6‐mm full‐width at half‐maximum Gaussian kernel.

### 2.6 Calculation of FDG subject residual profile

The subject residual profile (SRP) is a data‐driven normalization strategy originally introduced in the scaled subprofile model by Moeller et al.^30^ It converts the FDG‐PET uptake into residual maps that capture each participant's region‐relative metabolic deviations. SRP applies two successive centering operations: (i) row centering, which subtracts a subject's global mean uptake; and (ii) column centering, which subtracts the cohort‐wide voxel mean. Accordingly, voxels with positive SRP values are relatively hypermetabolic for that individual, whereas negative values indicate relative hypometabolism.

SRP maps were generated from the entire study cohort (*n* = 503) as follows. Preprocessed, spatially normalized FDG‐SUVR volumes within GM mask were vectorized to form an N×V data matrix, where rows (N) correspond to subjects and columns (V) to voxels. Matrix entries were logarithmically transformed and row‐centered to remove the subject‐wise mean. The group mean profile (GMP) was then created by voxel‐wise averaging, and column centering by subtracting the GMP produced the SRP map:

$$\mathrm{SR}P_{sv}=\log\left(D_{sv}\right)-\mathrm{mea}n_{s}-\mathrm{GM}P_{v},$$

where $\mathrm{SR}P_{sv}$ represents the residual, $\log(D_{sv})$ is the logarithm of the original data, $\mathrm{mea}n_{s}$ is the mean across all voxels for subject s, and $\mathrm{GM}P_{v}$ is the group mean for voxel v. Finally, regional brain metabolism for both SUVR and SRP methods was extracted within the GM mask based on the Automated Anatomical Labeling atlas (version 3)^31^ using the highly deformable registration algorithm implemented in the ANTs software.^32^

### 2.7 Quality assurance for image processing

All MRI and PET images, along with the preprocessing results from the automated pipelines, were visually reviewed for quality assurance by three researchers (S.W.K., S.J., and B.S.Y.), who were blinded to the participant details.

### 2.8 Statistical analysis

Statistical analyses were conducted using R (version 4.2.1) for demographic and clinical data and MATLAB (MathWorks, Natick, MA) with the SurfStat toolbox for voxel‐wise imaging analyses. Demographic and clinical characteristics were compared among the NC, AD^SAA−^, and AD^SAA+^ groups using one‐way ANOVA and *χ* ^2^ tests. To examine brain metabolic patterns according to SAA positivity, voxel‐wise general linear models (GLMs) were applied, controlling for age, sex, and education (Model 1), and additionally for the log‐transformed CSF pTau_181_/Aβ_42_ ratio (Model 2) to account for AD pathological burden. Region of interest (ROI)‐based analyses were then performed to identify regions showing the largest metabolic differences between AD^SAA−^ and AD^SAA+^ groups, from which the SRP‐max and SUVR‐max ROIs were defined. Subsequent GLMs examined the independent and combined effects of SAA positivity and AD pathology (reflected by the log‐transformed CSF pTau_181_/Aβ_42_ ratio) on these ROIs. To assess cognitive correlates, GLMs tested whether SRP‐max and SUVR‐max ROIs predicted annual cognitive change and cognitive variability, adjusting for demographics and baseline scores. Model‐fit comparisons using Akaike information criterion (AIC) and likelihood ratio tests evaluated whether these ROIs provided additional predictive value beyond SAA positivity and AD signature metabolism (Figure S2). The definition of AD signature metabolism is provided in the Supplementary Methods. Sensitivity analyses included (1) repeating the main analyses stratified by disease stage (MCI vs dementia); (2) substituting amyloid PET‐based CL values for CSF pTau_181_/Aβ_42_ ratio; and (3) employing linear mixed‐effects models (LMMs) to model longitudinal trajectories and intraindividual cognitive variability. Detailed modeling specifications for main and sensitivity analyses are described in the Supplementary Methods.

## 3 RESULTS

### 3.1 Study participants

Table 1 presents demographic and clinical characteristics of study participants. The three groups had comparable mean age and sex proportions. The AD^SAA−^ group had fewer years of education than the NC group but a comparable number of years to the AD^SAA+^ group. The three groups had comparable proportions of tremor, whereas the AD^SAA+^ group exhibited a higher proportion of gait disturbance compared to the AD^SAA−^ group. Compared to the NC group, both the AD^SAA−^ and AD^SAA+^ groups exhibited a higher proportion of *APOE4* carriers, shorter follow‐up durations, worse clinical outcomes, including baseline scores and annual rates of decline, and higher CSF pTau_181_/Aβ_42_ ratio levels. Compared to the AD^SAA−^ group, the AD^SAA+^ group had a higher proportion of patients with dementia and worse clinical outcomes, except for baseline visuospatial scores. The AD^SAA−^ and AD^SAA+^ groups had comparable proportions of *APOE4* carriers and similar CSF pTau_181_/Aβ_42_ ratio levels. The three groups had a comparable proportion of participants who underwent amyloid PET. Mean CL values were higher in both the AD^SAA−^ and AD^SAA+^ groups than in the NC group, with no significant difference between the two disease groups.

**TABLE 1:** Demographics and clinical characteristics of study participants.

	NC	AD^SAA−^	AD^SAA+^	*P*	*P* ^*^
Number	108	271	124	–	–
Age, year	73.0 ± 6.7	74.1 ± 7.5	75.3 ± 7.4	0.057	0.138
Female, *N* (%)	57 (52.8%)	113 (41.7%)	53 (42.7%)	0.135	0.846
Education, year	16.6 ± 2.7	15.8 ± 2.7	16.0 ± 2.9	**0.041**	0.571
Tremor, *N* (%)	14 (13%)	43 (15.9%)	19 (15.3%)	0.774	0.889
Gait disturbance, *N* (%)	12 (11.1%)	30 (11.1%)	23 (18.5%)	0.099	**0.040**
Stage	–	–	–	**<0.001**	**<0.001**
CU	108 (100%)	0 (0%)	0 (0%)	–	–
MCI	0 (0%)	149 (55%)	45 (36.3%)	–	–
Dementia	0 (0%)	122 (45%)	79 (63.7%)	–	–
APOE ε4	26 (24.1%)	188 (69.4%)	90 (72.6%)	**<0.001**	0.513
Baseline MMSE	29.1 ± 1.3	25.2 ± 3.7	23.8 ± 3.9	**<0.001**	**<0.001**
Baseline ADAS	8.80 ± 4.58	21.96 ± 9.26	25.29 ± 10.35	**<0.001**	**0.001**
Baseline memory	1.25 ± 0.77	−0.30 ± 0.81	−0.55 ± 0.87	**<0.001**	**0.005**
Baseline language	0.84 ± 0.78	−0.11 ± 0.90	−0.33 ± 0.85	**<0.001**	**0.018**
Baseline visuospatial	0.19 ± 0.60	−0.30 ± 0.93	−0.42 ± 0.95	**<0.001**	0.228
Baseline executive	0.65 ± 0.69	−0.40 ± 0.96	−0.74 ± 0.95	**<0.001**	**0.001**
Follow‐up duration	5.93 ± 3.63	3.26 ± 2.75	2.58 ± 2.21	**<0.001**	**0.034**
MMSE change	−0.05 ± 0.69	−1.49 ± 2.05	−2.36 ± 3.13	**<0.001**	**<0.001**
ADAS change	−0.32 ± 2.79	3.02 ± 5.51	5.24 ± 6.69	**<0.001**	**<0.001**
Memory change	−0.01 ± 0.22	−0.23 ± 0.37	−0.43 ± 0.44	**<0.001**	**<0.001**
Language change	−0.003 ± 0.31	−0.21 ± 0.56	−0.40 ± 0.49	**<0.001**	**0.001**
Visuospatial change	0.06 ± 0.48	−0.18 ± 0.74	−0.37 ± 0.79	**<0.001**	**0.014**
Executive change	0.04 ± 0.23	−0.25 ± 0.47	−0.36 ± 0.51	**<0.001**	**0.033**
pTau_181_/Aβ_42_ ratio	0.013 ± 0.004	0.061 ± 0.028	0.062 ± 0.029	**<0.001**	0.538
Amyloid PET, *N* (%)	92 (85.2%)	210 (77.5%)	97 (78.2%)	0.234	0.877
Centiloids	3.9 ± 16.9	83.0 ± 36.4	90.5 ± 39.0	**<0.001**	0.110

*Note*: Data are expressed as means ± standard deviations for continuous variables and as numbers (%) for categorical variables. Three‐group comparisons were performed using ANOVA or chi‐squared tests, as appropriate. Bolded P‐values indicate statistically significant difference at P < 0.05.

Abbreviations: AD, Alzheimer's disease; ADAS, Alzheimer's Disease Assessment Scale; CU, cognitively unimpaired; MCI, mild cognitive impairment; MMSE, Mini‐Mental State Examination; NC, normal control; PET, positron emission tomography; SAA, seed amplification assays.

* Post hoc comparisons between the AD^SAA−^ and AD^SAA+^ groups were conducted using chi‐square tests or independent *t*‐tests.

### 3.2 Comparisons of brain metabolism between NC, AD^SAA−^, and AD^SAA+^ groups

**FIGURE 1:**
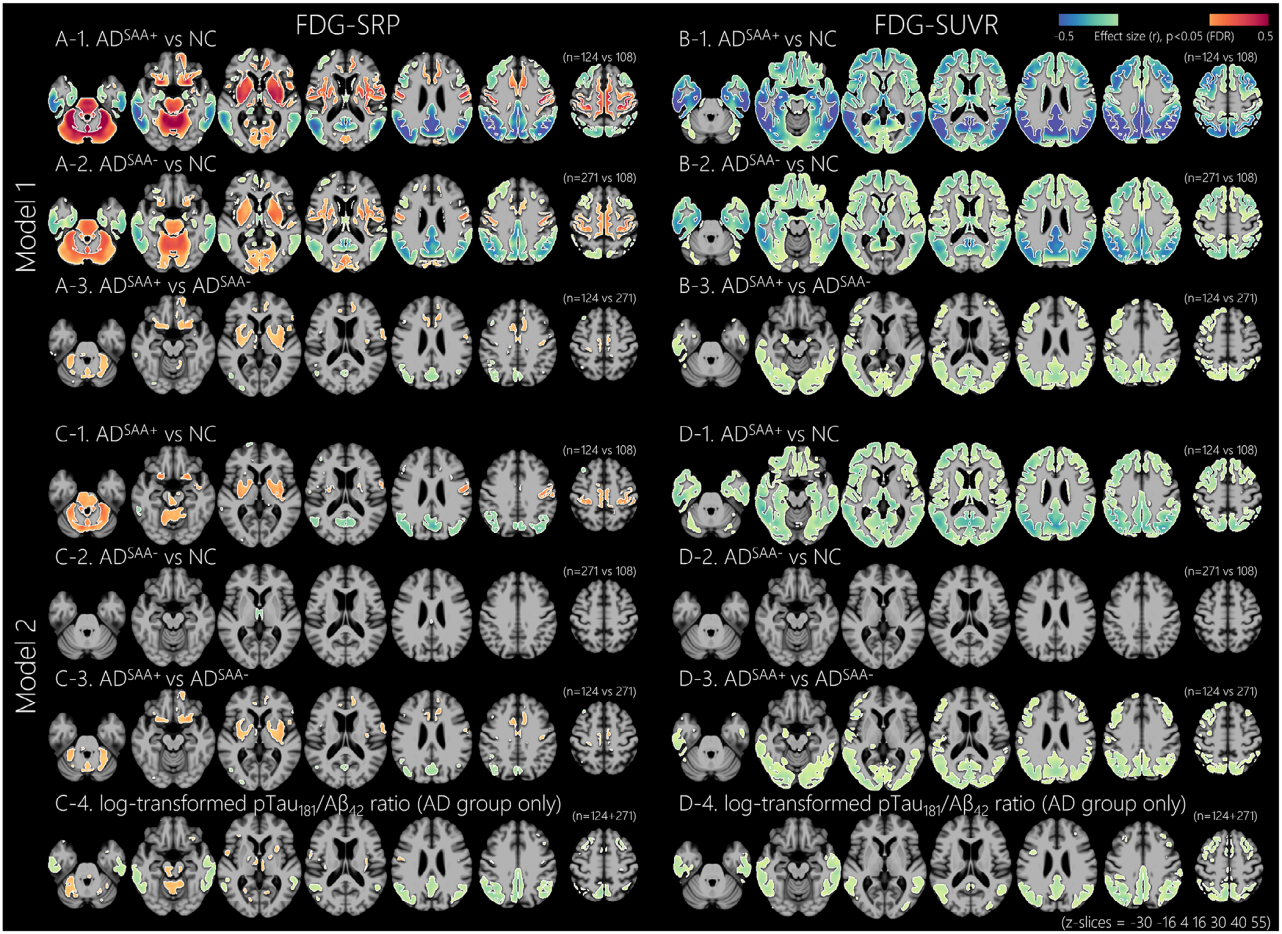
Effects of disease group and CSF AD biomarker on regional brain metabolism. Voxel‐wise analyses of FDG metabolism were performed using general linear models adjusted for age, sex, and education. Model 1 included disease group as a predictor (Panels A and B for FDG‐SRP and FDG‐SUVR, respectively), and Model 2 included both disease group and log‐transformed CSF pTau_181_/Aβ_42_ ratio as predictors (Panels C and D for FDG‐SRP and FDG‐SUVR, respectively). In group comparisons (A, B, C1 to C3, and D1 to D3), red areas show higher metabolism in the first group, and blue areas show lower metabolism. In correlation maps (C4, D4), which include only the AD group, red areas show positive correlations, and blue areas show negative correlations. Displayed results are thresholded at FDR‐corrected *p* < 0.05. Aβ, amyloid beta; AD, Alzheimer's disease; CSF, cerebrospinal fluid; FDG, ^18^F‐fluorodeoxyglucose; FDR, false discovery rate; MCI, mild cognitive impairment; NC, normal control; pTau_181_, tau phosphorylated at threonine 181; SAA, alpha‐synuclein seed amplification assay; SRP, subject residual profile; SUVR, standardized uptake value ratio.

Figure 1 illustrates the voxel‐wise comparison of brain metabolism among the three groups using the SRP and SUVR methods. In the Model 1 GLMs for FDG‐SRP, both the AD^SAA−^ and AD^SAA+^ groups, compared to the NC group, exhibited increased metabolism in the cerebellum, pons, midbrain, basal ganglia, thalamus, insula, anterior cingulate cortex, paracentral lobules, medial occipital cortices, orbitofrontal cortices, and primary sensorimotor cortices, while exhibiting decreased metabolism in the temporo‐parietal cortices and dorsolateral prefrontal cortices (Figure 1A[1,2]). Compared to the AD^SAA−^ group, the AD^SAA+^ group exhibited increased metabolism in the cerebellum, orbitofrontal cortices, basal ganglia, and anterior and middle cingulate cortices, while showing decreased metabolism in focal regions of the angular gyrus, cuneus, and occipital cortices (Figure 1A[3]). In ROI‐based analyses, the largest difference between the AD^SAA+^ and AD^SAA−^ groups was observed in the right putamen (left column in Figure S3). The Model 2 GLMs for FDG‐SRP showed that the AD^SAA+^ group had increased metabolism in the bilateral posterior putamen, dorsal cerebellum, pons, orbitofrontal cortices, and primary sensorimotor cortices compared to both the NC (Figure 1C[1]) and AD^SAA−^ groups (Figure 1C[3]), even after controlling for AD pathologic burden reflected by the log‐transformed CSF pTau_181_/Aβ_42_ ratio. A higher log‐transformed CSF pTau_181_/Aβ_42_ ratio was associated with decreased metabolism in the bilateral temporo‐parietal cortices and increased metabolism in the dorsal cerebellum (Figure 1C[4]). In contrast, the AD^SAA−^ group showed no significant metabolic differences compared to the NC group (Figure 1C[2]).

The Model 1 GLMs for FDG‐SUVR showed that, compared to the NC group, both the AD^SAA−^ and AD^SAA+^ groups exhibited decreased metabolism across widespread GM regions, except in the cerebellum, pons, midbrain, primary sensorimotor cortices, and medial occipital cortices (Figure 1B[1,2]). The AD^SAA+^ group also showed additional hypometabolism in the occipital regions compared to the NC group (Figure 1B[1]). Compared to the AD^SAA−^ group, the AD^SAA+^ group exhibited decreased metabolism in the widespread temporo‐parieto‐occipital cortices and focal regions of the dorsolateral prefrontal cortex (Figure 1B[3]). In ROI‐based analyses, the largest difference between the AD^SAA+^ and AD^SAA−^ groups was observed in the left middle occipital gyrus (right column in Figure S3). The Model 2 GLMs for FDG‐SUVR showed that, similar to the findings in the Model 1 GLMs, the AD^SAA+^ group exhibited decreased metabolism in widespread GM regions compared to the NC group (Figure 1D[1]), while the AD^SAA−^ group did not exhibit significant metabolic differences compared to the NC group (Figure 1D[2]). Compared to the AD^SAA−^ group, the AD^SAA+^ group exhibited decreased metabolism in the widespread temporo‐parieto‐occipital cortices and focal regions of the dorsolateral prefrontal cortices (Figure 1D[3]). Higher log‐transformed CSF pTau_181_/Aβ_42_ ratio was associated with decreased metabolism in the bilateral temporo‐parietal cortices and focal regions of the dorsolateral prefrontal cortices (Figure 1D[4]).

### 3.3 Association between right putamen SRP and left middle occipital gyrus SUVR

Table S1 presents the associations between left middle occipital gyrus SUVR and right putamen SRP in the NC, AD^SAA−^, AD^SAA+^ groups, as well as in the combined AD^SAA−^/AD^SAA+^ group and the entire cohort. Right putamen SRP was negatively associated with left middle occipital gyrus SUVR in both the AD^SAA−^ and AD^SAA+^ groups, but not in the NC group. In the combined AD^SAA−^/AD^SAA+^ group, an interaction effect between SAA positivity and left middle occipital gyrus SUVR on right putamen SRP was observed, indicating a stronger association in the AD^SAA+^ group than in the AD^SAA−^ group. In the entire cohort, the interaction effect of SAA positivity and left middle occipital gyrus SUVR was significant, whereas the interaction effect of pTau_181_/Aβ_42_ ratio levels and left middle occipital gyrus SUVR was not, suggesting that SAA positivity modified the association between left middle occipital gyrus SUVR and right putamen SRP, while pTau_181_/Aβ_42_ ratio levels did not.

### 3.4 Associations of SAA positivity and log‐transformed CSF pTau_181_/Aβ_42_ ratio with right putamen SRP or left middle occipital gyrus SUVR

**FIGURE 2:**
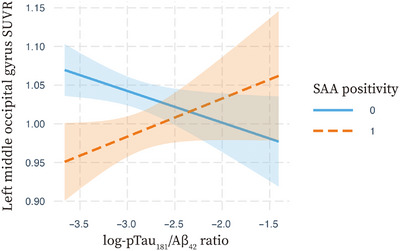
Interaction of SAA positivity and log‐transformed CSF pTau_181_/Aβ_42_ ratio on left middle occipital gyrus SUVR in AD participants. The results are based on the general linear models for left middle occipital SUVR after controlling for age, sex, education, and baseline Mini‐Mental State Examination score, separately performed in SAA‐positive and SAA‐negative AD participants. The standardized beta was 0.15 (*p* = 0.094) in the SAA‐positive group and −0.10 (*p* = 0.087) in the SAA‐negative group. AD, Alzheimer's disease; CSF, cerebrospinal fluid; log‐pTau_181_/Aβ_42_ ratio, log‐transformed phosphorylated tau/Aβ42 ratio; SAA, seed amplification assays; SUVR, standardized uptake value ratio.

Table 2 presents the independent and interaction effects of SAA positivity and log‐transformed CSF pTau_181_/Aβ_42_ ratio on right putamen SRP and left middle occipital gyrus SUVR in the entire cohort and the combined AD^SAA−^/AD^SAA+^ group. In the entire cohort, SAA positivity and higher log‐transformed CSF pTau_181_/Aβ_42_ ratio were independently associated with higher right putamen SRP and lower left middle occipital gyrus SUVR. An antagonistic interaction effect between SAA positivity and log‐transformed CSF pTau_181_/Aβ_42_ ratio was observed for left middle occipital gyrus SUVR, but not for right putamen SRP. Subgroup analyses showed that higher log‐transformed CSF pTau_181_/Aβ_42_ ratio was associated with lower left middle occipital gyrus SUVR in the SAA‐negative group, but not in the SAA‐positive group (data not shown). In the combined AD^SAA−^/AD^SAA+^ group, SAA positivity was associated with higher right putamen SRP and lower left middle occipital gyrus SUVR, whereas log‐transformed CSF pTau_181_/Aβ_42_ ratio was not. Similarly, an antagonistic interaction effect between SAA positivity and log‐transformed CSF pTau_181_/Aβ_42_ ratio was observed for left middle occipital gyrus SUVR, but not for right putamen SRP. Subgroup analyses showed that higher log‐transformed CSF pTau_181_/Aβ_42_ ratio was associated with lower left middle occipital gyrus SUVR in the SAA‐negative group, whereas, in the SAA‐positive group, it was associated with increased left middle occipital gyrus SUVR (Figure 2).

**TABLE 2:** Effect of SAA positivity and log‐transformed cerebrospinal fluid pTau_181_/Aβ_42_ ratio on right putamen SRP or left middle occipital gyrus SUVR.

	SAA positivity		log‐pTau_181_/Aβ_42_ ratio		Interaction	
	*β*	*P*	*β*	*P*	*β*	*P*
Whole participants						
Rt PUT SRP	0.12	**0.003**	0.14	**0.002**	–	–
Lt MOG SUVR	−0.15	**<0.001**	−0.11	**0.019**	–	–
Lt MOG SUVR	0.53	**0.039**	−0.15	**0.002**	0.69	**0.007**
AD participants						
Rt PUT SRP	0.13	**0.005**	0.04	0.366	–	–
Lt MOG SUVR	−0.16	**<0.001**	−0.04	0.413	–	–
Lt MOG SUVR	0.71	**0.029**	−0.12	**0.030**	0.87	**0.007**

*Note*: The results are based on general linear models adjusted for age, sex, education, and baseline MMSE score. SAA‐positivity and log‐transformed CSF pTau_181_/Aβ_42_ ratio were included as predictors. Interaction terms between SAA‐positivity and log‐transformed CSF pTau_181_/Aβ_42_ ratio were included in the model when statistically significant. P‐values in bold denote statistical significance at the P < 0.05 level.

Abbreviations: AD, Alzheimer's disease; *β*, standardized beta coefficient; log‐pTau_181_/Aβ_42_ ratio, log‐transformed phosphorylated tau 181/Aβ42 ratio; Lt MOG SUVR, left middle occipital gyrus metabolism assessed by standardized uptake value ratio; Rt PUT SRP, right putamen metabolism assessed by single residual profile method; SAA, seed amplification assays.

### 3.4 Associations of right putamen SRP and left middle occipital gyrus SUVR with cognitive decline and variability

Table 3 presents the univariable and multivariable effects of right putamen SRP and left middle occipital gyrus SUVR on cognitive outcomes, analyzed separately in the NC, AD^SAA−^, and AD^SAA+^ groups. In the NC group, neither right putamen SRP nor left middle occipital gyrus SUVR showed any association with cognitive decline or variability.

**TABLE 3:** Association between right putamen SRP or left middle occipital gyrus SUVR and rate/variability of cognitive changes

	NC				AD^SAA−^				AD^SAA+^			
	Rt PUT SRP		Lt MOG SUVR		Rt PUT SRP		Lt MOG SUVR		Rt PUT SRP		Lt MOG SUVR	
	*β*	P	*β*	P	*β*	P	*β*	P	*β*	P	*β*	P
**Univariable model**												
*Annual change*												
MMSE	−0.02	0.857	0.09	0.347	−0.29	**<0.001**	0.26	**<0.001**	−0.27	**0.003**	0.16	0.073
ADAS	−0.19	0.075	0.02	0.795	0.21	**0.002**	−0.18	**0.007**	0.37	**<0.001**	−0.21	0.022
Memory	0.11	0.325	−0.21	0.022	−0.14	0.040	0.24	**<0.001**	−0.08	0.450	0.03	0.787
Language	−0.17	0.129	−0.07	0.488	−0.12	0.085	0.17	**0.012**	−0.25	**0.010**	0.07	0.439
Visuospatial	−0.04	0.740	0.01	0.943	−0.15	**0.021**	0.13	0.055	−0.15	0.127	0.17	0.071
Executive	−0.02	0.840	0.11	0.250	−0.12	0.068	0.22	**0.001**	−0.13	0.225	0.18	0.076
*Variability*												
MMSE	−0.02	0.892	0.17	0.077	0.23	**0.001**	−0.30	**<0.001**	0.33	**0.001**	−0.25	**0.010**
ADAS	−0.08	0.438	−0.04	0.623	0.23	**0.001**	−0.22	**0.001**	0.35	**<0.001**	−0.39	**<0.001**
Memory	0.14	0.200	−0.09	0.301	0.17	**0.013**	−0.21	**0.001**	0.18	0.070	−0.18	0.057
Language	0.09	0.437	0.06	0.509	0.12	0.096	−0.19	**0.004**	0.25	**0.009**	−0.17	0.061
Visuospatial	0.15	0.179	0.13	0.143	0.12	0.065	−0.15	**0.024**	0.15	0.123	−0.28	**0.004**
Executive	0.13	0.263	0.04	0.704	0.08	0.272	−0.25	**<0.001**	0.12	0.275	−0.21	0.036
**Multivariable model**												
*Annual change*												
MMSE	−0.02	0.879	0.09	0.351	−0.24	**<0.001**	0.21	**0.001**	−0.24	**0.015**	0.07	0.464
ADAS	−0.18	0.078	0.01	0.897	0.18	**0.010**	−0.15	**0.032**	0.33	**0.001**	−0.09	0.354
Memory	0.09	0.384	−0.21	0.026	−0.09	0.176	0.22	**0.001**	−0.08	0.482	0.00	0.975
Language	−0.18	0.121	−0.08	0.435	−0.09	0.215	0.15	**0.028**	−0.26	**0.014**	−0.02	0.827
Visuospatial	−0.04	0.743	0.00	0.957	−0.13	0.059	0.09	0.169	−0.10	0.342	0.14	0.177
Executive	−0.02	0.885	0.11	0.256	−0.08	0.218	0.20	**0.003**	−0.08	0.488	0.16	0.144
*Variability*												
MMSE	−0.01	0.899	0.17	0.079	0.17	**0.012**	−0.26	**<0.001**	0.27	**0.009**	−0.14	0.170
ADAS	−0.09	0.417	−0.05	0.582	0.19	**0.006**	−0.18	**0.006**	0.22	0.022	−0.31	**0.001**
Memory	0.13	0.222	−0.09	0.335	0.13	0.060	−0.19	**0.005**	0.13	0.246	−0.13	0.195
Language	0.09	0.417	0.07	0.483	0.08	0.267	−0.17	**0.010**	0.21	0.038	−0.10	0.334
Visuospatial	0.15	0.151	0.14	0.122	0.09	0.189	−0.12	0.064	0.06	0.565	−0.26	0.012
Executive	0.13	0.255	0.04	0.656	0.03	0.679	−0.25	**<0.001**	0.05	0.645	−0.20	0.066

*Note*: The results are based on general linear models using right putamen SRP or left middle occipital gyrus SUVR as predictors. The univariable model included either right putamen SRP or left middle occipital gyrus SUVR as a predictor, whereas the multivariable model includes both as predictors. Covariates include age, sex, education, and baseline cognitive scores (e.g., baseline MMSE, baseline ADAS). Multiple comparisons were corrected using the FDR method across 12 cognitive outcomes (6 annual change outcomes + 6 variability outcomes) within each group. The *p*‐values reported are uncorrected, and those that remained significant after FDR correction are shown in bold.

Abbreviations: AD, Alzheimer's disease; ADAS, Alzheimer's Disease Assessment Scale; *β*, standardized beta coefficient; FDR, false discovery rate; Lt MOG SUVR, left middle occipital gyrus metabolism assessed by standardized uptake value ratio; MMSE, Mini‐Mental State Examination; NC, normal control; Rt PUT SRP, right putamen metabolism assessed by subject residual profile method; SAA, seed amplification assays.

In the AD^SAA−^ group, univariable models for the rate of cognitive changes showed that higher right putamen SRP was associated with faster cognitive decline in MMSE, ADAS, and visuospatial scores, while lower left middle occipital gyrus SUVR was associated with faster decline in MMSE, ADAS, memory, language, and executive scores. Multivariable models yielded similar results, except for the visuospatial scores, where the effect of right putamen SRP was no longer significant. Univariable models for cognitive variability in the AD^SAA−^ group showed that higher right putamen SRP was associated with increased variability in MMSE, ADAS, and memory scores, while lower middle occipital gyrus SUVR was associated with increased variability across all cognitive outcomes. Multivariable models produced similar findings, with two exceptions: for memory scores, lower left middle occipital gyrus SUVR mediated the effect of right putamen SRP; and for visuospatial scores, the effect of left middle occipital gyrus SUVR was no longer significant.

In the AD^SAA+^ group, both univariable and multivariable models for the rate of cognitive worsening showed that higher right putamen SRP was associated with faster decline in the MMSE, ADAS, and language scores, whereas left middle occipital gyrus SUVR showed no significant association. Univariable models for cognitive variability demonstrated that higher right putamen SRP was associated with increased variability in MMSE, ADAS, and language scores, while lower middle occipital gyrus SUVR was associated with greater variability in MMSE, ADAS, and executive scores. Multivariable models yielded similar findings: Higher right putamen SRP remained significantly associated with increased MMSE variability and showed a trend toward association with ADAS variability (adjusted *p* = 0.052). In contrast, lower left middle occipital gyrus SUVR was not independently associated with MMSE variability but was associated with greater variability in ADAS scores.

### 3.5 Associations of SAA positivity, right putamen SRP, and AD signature metabolism with cognitive decline and variability

In GLM 1, SAA positivity was associated with faster worsening across all cognitive measures except for the executive score, as well as with greater MMSE variability (Table 4). In GLM 2, which additionally included right putamen SRP, higher right putamen SRP was associated with faster worsening across all cognitive measures except for the memory score, as well as with greater variability in MMSE, ADAS, memory, and language scores. After adjusting for right putamen SRP, SAA positivity was only associated with faster decline in memory and language scores. Across all six cognitive decline measures and six cognitive variability measures, GLM 2 demonstrated consistently lower AICs compared to GLM 1. Statistically significant improvements in model fit were observed for most outcomes, except for memory decline and variability in visuospatial and executive scores. In GLM 3, which included SAA positivity and AD signature metabolism, more severe AD hypometabolism was associated with faster worsening and greater variability across all cognitive measures, whereas SAA positivity was only associated with faster memory decline. In GLM 4, which further included right putamen SRP, more severe AD hypometabolism was associated with greater longitudinal worsening across all cognitive measures, while SAA positivity was associated with faster memory decline. Furthermore, higher right putamen SRP was associated with faster worsening and greater variability in MMSE and ADAS scores. For MMSE and ADAS, GLM 4 demonstrated lower AICs than GLM 3, and these improvements in model fit were statistically significant.

**TABLE 4:** Effects of SAA positivity, right putamen subject residual profile, and Alzheimer's disease (AD) signature metabolism on the rate/variability of cognitive changes in AD participants.

Predictors	SAA positivity		Rt PUT SRP		AD signature metabolism				
Outcomes	*β*	*p*	*β*	*p*	*β*	*p*	AIC	*p* ^1 vs 2^	*p* ^3 vs 4^
**GLM 1**									
*Annual change*									
MMSE	−0.13	**0.013**	–	–	–	–	1686.69	**<0.001**	–
ADAS	0.16	**0.002**	–	–	–	–	2328.09	**<0.001**	–
Memory	−0.22	**<0.001**	–	–	–	–	360.22	0.048	–
Language	−0.17	**0.001**	–	–	–	–	595.60	**0.004**	–
Visuospatial	−0.13	**0.010**	–	–	–	–	836.77	**0.011**	–
Executive	−0.11	0.035	–	–	–	–	490.34	**0.015**	–
*Variability*									
MMSE	0.14	**0.010**	–	–	–	–	1352.10	**<0.001**	–
ADAS	0.11	0.034	–	–	–	–	1937.54	**<0.001**	–
Memory	0.09	0.096	–	–	–	–	29.13	**0.002**	–
Language	0.07	0.214	–	–	–	–	116.26	**0.003**	–
Visuospatial	0.10	0.058	–	–	–	–	192.69	0.051	–
Executive	0.04	0.407	–	–	–	–	35.96	0.126	–
**GLM 2**									
*Annual change*									
MMSE	−0.09	0.080	−0.27	**<0.001**	–	–	1661.41	**<0.001**	–
ADAS	0.13	0.013	0.26	**<0.001**	–	–	2307.56	**<0.001**	–
Memory	−0.20	**<0.001**	−0.11	0.048	–	–	358.23	0.048	–
Language	−0.14	**0.007**	−0.16	**0.004**	–	–	589.14	**0.004**	–
Visuospatial	−0.11	0.037	−0.14	**0.011**	–	–	832.14	**0.011**	–
Executive	−0.09	0.078	−0.14	**0.015**	–	–	486.24	**0.015**	–
*Variability*									
MMSE	0.10	0.056	0.27	**<0.001**	–	–	1330.50	**<0.001**	–
ADAS	0.08	0.144	0.27	**<0.001**	–	–	1915.83	**<0.001**	–
Memory	0.06	0.241	0.17	**0.002**	–	–	21.30	**0.002**	–
Language	0.04	0.443	0.17	**0.003**	–	–	109.40	**0.003**	–
Visuospatial	0.08	0.130	0.11	0.051	–	–	190.81	0.051	–
Executive	0.03	0.540	0.09	0.126	–	–	35.57	0.126	–
**GLM 3**									
*Annual change*									
MMSE	−0.08	0.098	–	–	0.35	**<0.001**	1646.00	–	**<0.001**
ADAS	0.12	0.014	–	–	−0.31	**<0.001**	2300.68	–	**<0.001**
Memory	−0.18	**<0.001**	–	–	0.27	**<0.001**	339.88	–	0.434
Language	−0.13	0.011	–	–	0.24	**<0.001**	579.39	–	0.068
Visuospatial	−0.09	0.087	–	–	0.26	**<0.001**	816.59	–	0.246
Executive	−0.07	0.189	–	–	0.32	**<0.001**	461.93		0.250
*Variability*									
MMSE	0.08	0.099	–	–	−0.39	**<0.001**	1305.17	–	**0.002**
ADAS	0.06	0.237	–	–	−0.44	**<0.001**	1880.17	–	**0.002**
Memory	0.04	0.410	–	–	−0.32	**<0.001**	−0.49	–	0.079
Language	0.03	0.605	–	–	−0.26	**<0.001**	97.70	–	0.068
Visuospatial	0.07	0.175	–	–	−0.15	**0.006**	187.01	–	0.253
Executive	0.002	0.974	–	–	−0.32	**<0.001**	9.70	–	0.803
**GLM 4**									
*Annual change*									
MMSE	−0.06	0.207	−0.19	**<0.001**	0.29	**<0.001**	1634.65	–	**<0.001**
ADAS	0.10	0.036	0.19	**<0.001**	−0.25	**<0.001**	2290.09	–	**<0.001**
Memory	−0.18	**0.001**	−0.04	0.434	0.25	**<0.001**	341.26	–	0.434
Language	−0.12	0.021	−0.10	0.068	0.21	**<0.001**	577.98	–	0.068
Visuospatial	−0.08	0.122	−0.07	0.246	0.23	**<0.001**	817.21	–	0.246
Executive	−0.06	0.234	−0.07	0.250	0.31	**<0.001**	462.57	–	0.250
*Variability*									
MMSE	0.07	0.181	0.17	**0.002**	−0.34	**<0.001**	1297.64	–	**0.002**
ADAS	0.04	0.402	0.17	**0.002**	−0.39	**<0.001**	1871.99	–	**0.002**
Memory	0.03	0.541	0.10	0.079	−0.29	**<0.001**	−1.64	–	0.079
Language	0.02	0.766	0.10	0.068	−0.23	**<0.001**	96.29	–	0.068
Visuospatial	0.06	0.230	0.07	0.253	−0.13	**0.025**	187.68	–	0.253
Executive	0.0004	0.994	0.01	0.803	−0.31	**<0.001**	11.64	–	0.803

*Note*: The results are based on general linear model for the rate/variability of cognitive changes. GLM 1 included SAA positivity; GLM 2 included SAA positivity and right putamenSRP; GLM 3 included SAA positivity and AD signature metabolism; and GLM 4 included all three predictors. All models were adjusted for baseline cognitive performance, age, sex, and education. *p*‐Values for model comparison were obtained from likelihood ratio tests comparing GLM 1 vs. GLM 2 (*p* ^1 vs. 2^) and GLM 3 vs. GLM 4 (*p* ^3 vs. 4^), respectively. Multiple comparisons were corrected using the false discovery rate (FDR) across 12 cognitive outcomes. The *p*‐values reported are uncorrected, and those that remained significant after FDR correction are shown in bold.

Abbreviations: AD, Alzheimer's disease; ADAS, Alzheimer's Disease Assessment Scale; AIC, Akaike information criterion; *β*, standardized beta coefficient; FDR, false discovery rate; MMSE, Mini‐Mental State Examination; Rt PUT SRP, right putamen metabolism assessed by subject residual profile method; SAA, seed amplification assays.

When substituting left middle occipital gyrus SUVR for right putamen SRP (Table S2), the models including both SAA positivity and left middle occipital gyrus SUVR demonstrated a better fit compared to the models using SAA positivity alone. However, the inclusion of the left middle occipital gyrus SUVR in the model containing both SAA positivity and AD signature metabolism did not result in further improvements in model fit.

### 3.6 Sensitivity analyses

Sensitivity analyses stratified by disease stage (MCI and dementia) yielded results largely consistent with the main findings (Figure S4 and Table S3). Although the differences between the MCI with AD^SAA+^ (MCI^SAA+^) and MCI with AD^SAA−^ (MCI^SAA−^), as well as between the dementia with AD^SAA+^ (Dementia^SAA+^) and dementia with AD^SAA−^ (Dementia^SAA−^) groups, reached significance only at the uncorrected threshold (*p* < 0.05), perhaps due to reduced sample sizes, the AD^SAA+^ group consistently showed increased right putamen SRP and decreased left middle occipital gyrus SUVR after controlling for disease stage. Among these, only increased right putamen SRP remained significantly associated with global cognitive worsening in the AD^SAA+^ group. When SAA positivity and AD signature metabolism were jointly considered (Tables S4–S7), right putamen SRP was the only metabolic marker that significantly improved the explanation of global cognitive decline. Substituting the log‐transformed CSF pTau_181_/Aβ_42_ ratio with amyloid PET‐based CL values produced highly consistent results (Figure S5 and Table S8), further confirming the robustness of the main findings. LLMs for longitudinal cognitive decline (Table S9), LMM‐derived cognitive variability (Table S10), and LMMs assessing the additive effect of right putamen SRP beyond AD signature metabolism (Tables S11 and S12) yielded results consistent with GLM‐based analyses. Detailed results of these sensitivity analyses are provided in the Supplementary Results.

## 4 DISCUSSION

In this study, we investigated the associations between SAA positivity and brain metabolism using both SUVR and SRP methods, as well as the associations between metabolic changes and the rate and variability of cognitive decline in patients with AD. Our study produced several major findings. First, the AD^SAA+^ group exhibited putaminal hypermetabolism (identified using the SRP method) compared to the AD^SAA−^ group, along with occipital hypometabolism (identified using the SUVR method). Second, putaminal hypermetabolism was associated with both faster decline and greater variability in general cognition in both the AD^SAA−^ and AD^SAA+^ groups, with the AD^SAA+^ group additionally showing a faster decline in language function. Occipital hypometabolism was associated with faster decline and greater variability in cognitive function – except for visuospatial function – in the AD^SAA−^ group. In contrast, in the AD^SAA+^ group, occipital hypometabolism was not linked to faster cognitive decline but was associated with variability in ADAS scores. Third, in the combined group of AD^SAA−^ and AD^SAA+^, adding putaminal hypermetabolism to models, including SAA positivity and AD signature hypometabolism, better predicted faster cognitive decline and greater variability, whereas adding the occipital hypometabolism did not. Fourth, SAA positivity was associated with putaminal hypermetabolism regardless of CSF pTau_181_/Aβ_42_ ratio, whereas the association between CSF pTau_181_/Aβ_42_ ratio and occipital metabolism differed according to SAA status: A higher CSF pTau_181_/Aβ_42_ ratio was associated with higher occipital metabolism in SAA‐positive patients but with lower occipital metabolism in SAA‐negative patients. Taken together, our results suggest that putaminal hypermetabolism could serve as a more reliable and consistent biomarker for LB pathology among individuals with AD.

Consistent with previous studies,^10^, ^12^, ^13^ the AD^SAA+^ group exhibited posterior occipito‐parietal hypometabolism compared to the AD^SAA−^ group, as identified using conventional SUVR methods. In contrast, the SRP methods primarily detected hypermetabolism in the cerebellum, olfactory cortex, basal ganglia, and anterior cingulate cortex, along with occipital hypometabolism in the AD^SAA+^ group. When analyzed separately within each disease stage (MCI and dementia), the associations of SAA positivity with left middle occipital gyrus SUVR and right putamen SRP were significant only at the uncorrected threshold, likely due to reduced sample sizes (Figure S4). However, in the analysis including all AD participants with disease stage as a covariate, SAA positivity remained significantly associated with both metabolic indices (Figure S6). Previous studies have demonstrated that patients with LBD – including PD and DLB – exhibit hypermetabolism in the cerebellum, amygdala, hippocampus, putamen, and somatomotor cortices compared to controls.^15^, ^33^, ^34^, ^35^, ^36^ Furthermore, this hypermetabolism has been associated with disease severity, including reduced dopamine transporter uptake^18^, ^19^, ^37^ and worsening clinical symptoms.^34^, ^38^, ^39^, ^40^, ^41^ The discrepancy between SRP and SUVR methods in detecting LB pathology‐related hypermetabolism can be attributed to reference region issues in SUVR methods.

Conventional SUVR approaches typically rely on particular reference regions such as cerebellum or pons; however, these regions may exhibit increased metabolism in the presence of LB pathology.^36^, ^41^ In contrast, the SRP is a data‐driven global normalization method that models voxel‐level deviations without assuming any specific region is invariant. By capturing the overall metabolic distribution, SRP highlights localized metabolic abnormalities, making it less susceptible to biases arising from affected reference regions. Therefore, integrating both approaches provides complementary perspectives, thereby enhancing the robustness and interpretability of metabolic findings related to LB pathology. Considering that cerebellar GM metabolism measured by SRP is positively associated with basal ganglia SRP metabolism and negatively associated with posterior occipito‐parietal SRP metabolism (Figure S7), putaminal hypermetabolism observed in SRP tends to be underestimated in SUVR, whereas occipital hypometabolism in SRP tends to be overestimated. Note that the SRP represents deviations from a study‐specific metabolic distribution, thereby requiring careful interpretation as they reflect relative differences rather than indicating absolute metabolic levels.

Putaminal hypermetabolism was associated with faster decline in general cognition and greater variability in both the AD^SAA−^ and AD^SAA+^ groups, independent of occipital hypometabolism. In contrast, occipital hypometabolism was independently associated with faster cognitive decline and increased variability only in the AD^SAA−^ group. Recent neuropathological studies have shown that among LBD subtypes – including diffuse cortical, limbic‐transitional, and brainstem‐predominant forms – diffuse cortical LBD demonstrates nearly 100% sensitivity on the SAA test.^42^ However, SAA test sensitivity decreases to 35% to 65% when LB pathology is confined to the limbic‐transitional or brainstem regions.^42^, ^43^ Furthermore, SAA test sensitivity for amygdala‐predominant LB pathology ranges from 14.3% to 63.6%,^44^, ^45^, ^46^ suggesting that some individuals in the AD^SAA−^ group may still harbor LB pathology, with their occipital hypometabolism possibly reflecting underlying processes related to cognitive decline and fluctuation. Sensitivity analyses in the MCI stage showed that right putamen SRP, but not left middle occipital gyrus, predicted global cognitive decline, although this association was observed only in the AD^SAA+^ group (Table S3). Although putaminal hypermetabolism was not associated with cognitive decline, and hypometabolism in the left middle occipital gyrus predicted executive decline in the MCI^SAA−^ subgroup, the effect of left middle occipital gyrus SUVR on cognition was not observed in the Dementia^SAA−^ subgroup or in AD^SAA+^ individuals. Moreover, the association between SAA positivity and left middle occipital gyrus SUVR was moderated by AD biomarkers, showing an antagonistic interaction (Table 2 and Table S1). This suggests that putaminal hypermetabolism may improve the detection of LB pathology among individuals with AD.

In patients with AD, SAA positivity was associated with putaminal hypermetabolism independently of CSF pTau_181_/Aβ_42_ ratio, while a significant antagonistic interaction between SAA positivity and log‐transformed CSF pTau_181_/Aβ_42_ ratio on occipital hypometabolism was observed (Figure 2). Specifically, the interaction plot revealed that higher CSF pTau_181_/Aβ_42_ ratio was associated with higher occipital metabolism in the SAA‐positive group, whereas the opposite pattern was observed in the SAA‐negative group. To enhance clinical applicability, we conducted sensitivity analyses using amyloid PET‐based CL values instead of CSF pTau_181_/Aβ_42_ ratio (Table S8 and Figure S5). These analyses yielded consistent results, reinforcing the robustness of our findings. This observation is consistent with our previous study, which demonstrated that increased occipital metabolism could be driven by a higher burden of AD pathology in the presence of LB pathology.^47^ These complex relationships among AD pathology, LB pathology, and occipital metabolism may explain why occipital hypometabolism does not reliably predict cognitive decline and variability in the AD^SAA+^ group (Table 3). Furthermore, in the combined group of AD^SAA+^ and AD^SAA−^, putaminal hypermetabolism independently predicted faster cognitive decline and greater variability, and when added to the model, including SAA positivity and AD signature hypometabolism, it significantly improved model fit (Table 4 and Table S11). In contrast, occipital hypometabolism did not show such associations (Tables S2 and S12). Even when stratified in MCI and dementia stages, right putamen SRP still improved model fit for predicting general cognitive decline over SAA positivity and AD signature hypometabolism (Tables S4 and S5), while left middle occipital gyrus SUVR showed no such effect (Tables S6 and S7). These results suggest that putaminal hypermetabolism could serve as a consistent biomarker for detecting LB‐related features, regardless of SAA positivity or the burden of AD pathology.

This study has several limitations. First, although longitudinal cognitive data were used to assess annual rates of cognitive decline and its variability, the observational nature of the ADNI dataset limits the ability to establish causality between metabolic changes, SAA positivity, and cognitive outcomes. Second, the study relied on CSF‐based biomarkers without neuropathological confirmation, which may have led to misclassification of participants with respect to AD and LB pathology status. Third, the lack of detailed clinical assessments specific to LBD features – such as cognitive fluctuations, parkinsonism, rapid eye movement sleep behavior disorder, and visual hallucinations – limits our ability to correlate observed metabolic patterns with core clinical manifestations of LBD. Even though the AD^SAA+^ group had a higher proportion of gait disturbance compared with the AD^SAA−^ group, logistic regression analyses assessing the association between regional brain metabolism and the presence of gait disturbance, adjusted for age, sex, and education, revealed no significant findings across the entire cohort, the overall AD group (AD^SAA−^ + AD^SAA+^), or within each subgroup (AD^SAA−^ and AD^SAA+^) (data not shown). This lack of significant association could be attributed to several factors. First, parkinsonian features in the ADNI dataset were not systematically assessed using standardized motor examinations, and the available data were limited to binary clinical observations (i.e., presence or absence), which may not adequately capture the severity of parkinsonism. Second, cardinal motor features such as bradykinesia and rigidity were not documented in the dataset. Third, the older age of the AD^SAA+^ group may have contributed to the higher frequency of gait disturbance observed. Future studies are warranted to validate the utility of the SRP method across diverse clinical populations and to examine its potential for accurately predicting LBD‐related clinical symptoms. Fourth, although we used the standard deviation of longitudinal MMSE scores to capture intraindividual cognitive variability, this approach is inherently limited by the ceiling effect and the restricted variability of the MMSE, particularly in the less impaired population. Nevertheless, in more cognitively impaired individuals, variability was greater and may provide clinically meaningful information. Importantly, our sensitivity analyses using LMM‐derived variability measures yielded results consistent with those based on the standard deviation of MMSE, reinforcing the robustness of our findings. Moreover, the use of standard deviations of clinical measures is well established in dementia research – for example, HbA1c^48^ or glycemic variability^49^ in relation to disease progression.

Despite these limitations, our findings suggest that among patients within AD–LB spectrum, putaminal hypermetabolism identified using the SRP method better predicts cognitive decline and fluctuation compared to occipital hypometabolism assessed by the SUVR method. Striatal hypermetabolism detected using SRP methods may serve as a robust metabolic marker of LBD co‐pathology in AD.

## CONFLICT OF INTEREST STATEMENT

The authors have no conflicts of interest to disclose. Author disclosures are available in the Supporting Information.

## CONSENT STATEMENT

The need for informed consent was waived due to the retrospective nature of the study.

## Supporting information



Supporting Information

Supporting Information
