# Supplementary material for: Putaminal hypermetabolism identifies Lewy body co‐pathology in Alzheimer's disease
Source: Alzheimers Dement. 2025 Nov 22;21(11):e70920. doi: 10.1002/alz.70920 (PMC12639405; doi:10.1002/alz.70920)
Supplement: Supplementary file 1 — Supporting Information [file ALZ-21-e70920-s001.docx]

**Supplementary Methods**

*AD signature brain metabolism*

We defined the AD signature metabolism by performing voxel-wise comparisons of brain metabolism between individuals with AD and NCs. To identify increasingly stringent hypometabolic regions, we applied stepwise t-score thresholds ranging from 5.0 to 10.0 in increments of 0.5. For each threshold-defined region, we calculated the mean SUVR value and subsequently fitted linear mixed models predicting cognitive trajectories. The t-score threshold associated with the lowest AIC in these models was selected as the optimal definition of the AD signature metabolism. Among the tested thresholds, we selected -9 as it was the most frequently optimal or near-optimal across cognitive outcomes (MMSE: -9, ADAS: -9, Memory: -8.5, Language: -8.5, Visuospatial: -9.5, Executive: -9).

*Statistical analysis*

Statistical analyses of demographic and clinical data were conducted using the R statistical software (version 4.2.1) and voxel-wise imaging analyses were performed using MATLAB (MathWorks, Inc., Natick, MA, USA) and SurfStat toolbox (<http://www.math.mcgill.ca/keith/surfstat/>). One-way analysis of variance with post-hoc analyses and χ^2^ tests were used to compare the demographics and clinical features between the NC, AD^SAA−^, and AD^SAA+^ groups.

To examine the patterns of brain metabolism according to SAA positivity in patients with AD, voxel-wise brain metabolism was compared among the NC, AD^SAA−^, and AD^SAA+^ groups using a general linear model (GLM) controlling for age, sex, and education (Model 1). Subsequently, to rule out the possibility that the differences in brain metabolism among the three groups were attributable to the severity of AD pathology, an additional GLM controlling for the log-transformed CSF pTau181/Aβ42 ratio was performed (Model 2). CSF pTau_181_/Aβ_42_ ratio values were log-transformed due to their right-skewed distribution. The voxel-wise statistical outcomes, including effect sizes (r) within statistically significant areas (false discovery rate-corrected P < 0.05) were displayed in the neurological convention.

To identify brain regions showing the greatest metabolic difference between pure AD and mixed AD-LB, ROI-based analyses were performed on 95 ROIs to specifically compare the AD^SAA−^ and AD^SAA+^ groups after controlling for age, sex, and education (**Supplementary Figure 3**). Among the brain regions that showed significant differences in SRP and SUVR metabolic comparisons between the AD^SAA−^ and AD^SAA+^ groups, we selected the regions showing the greatest differences in each modality, designated as the SRP-max ROI and SUVR-max ROI, respectively. Subsequently, to determine the extent to which SAA positivity and AD pathological burden explained SRP-max ROI and SUVR-max ROI, GLMs were performed in the entire cohort and within AD participants (AD^SAA−^ + AD^SAA+^), with SAA positivity and the log-transformed CSF pTau_181_/Aβ_42_ ratio entered as predictors after controlling for age, sex, and education. Interaction terms were included in the GLM only when statistically significant. The inclusion of CN individuals provided a metabolic reference for interpreting group differences and enabled the investigation of early metabolic changes encompassing the transition from healthy aging to neurodegenerative disease.

To examine the extent to which the SRP-max and SUVR-max ROIs predict cognitive changes, GLMs for annual cognitive changes and cognitive variability were conducted using SRP-max ROI and SUVR-max ROI as predictors, adjusting for age, sex, education, and baseline scores, separately within the NC, AD^SAA−^ and AD^SAA+^ groups. Next, to evaluate whether the SRP-max ROI or SUVR-max ROI provides additional value in predicting cognitive changes among participants with AD, in addition to SAA positivity or AD signature metabolism, we conducted GLMs and compared model fit using both Akaike Information Criterion (AIC) and likelihood ratio tests. The comparisons were performed between the following pairs of models:

1. SAA-only model (*GLM 1*) vs. SAA + SRP-max ROI model (*GLM 2*), assessing whether adding SRP-max ROI improves prediction over SAA alone.
2. SAA-only model (*GLM 1*) vs. SAA + SUVR-max ROI model (*GLM 2*), assessing whether adding SUVR-max ROI improves prediction over SAA alone.
3. SAA + AD signature metabolism model (*GLM 3*) vs. SAA + AD signature metabolism + SRP-max ROI model (*GLM 4*), assessing whether SRP-max ROI adds further predictive value beyond SAA and AD signature metabolism.
4. SAA + AD signature metabolism model (*GLM 3*) vs. SAA + AD signature metabolism + SUVR-max ROI model (*GLM 4*), assessing whether SUVR-max ROI adds further predictive value beyond SAA and AD signature metabolism.

AD signature metabolism was derived from voxel-wise comparisons of brain metabolism between individuals with AD and NCs (**Supplementary Figure 2**), as detailed in the Supplementary Methods. Mean SUVR values within the threshold-defined hypometabolic regions were then extracted for each participant and used as predictors in the statistical models.

We performed the following sensitivity analyses. First, to examine whether the metabolic changes observed in the AD^SAA^^+^ and AD^SAA^^−^ groups differed by disease stage (MCI or dementia), we conducted GLMs comparing NC, MCI with AD^SAA+^ (MCI^SAA+^), and MCI with AD^SAA^^−^ (MCI^SAA−^), as well as those comparing NC, dementia with AD^SAA+^ (Dementia^SAA+^), and dementia with AD^SAA−^ (Dementia^SAA−^), adjusting for age, sex, and education. Second, among AD participants, GLMs for SRP and SUVR were conducted with SAA positivity and disease stage (MCI vs. dementia) as predictors, adjusting for age, sex, and education. Because all NC participants were SAA-negative, the NC group was excluded from this analysis. Third, the main analyses were stratified by disease stage (MCI and dementia) to examine (1) the extent to which SRP-max ROI and SUVR-max ROI predicted annual cognitive changes and cognitive variability, and (2) whether these indices provided additional predictive value for cognitive changes beyond SAA positivity and AD signature metabolism. Fourth, to assess the effects of SAA positivity and AD pathological burden on SRP-max ROI and SUVR-max ROI, we repeated the analyses using amyloid PET-based CL values instead of the log-transformed CSF pTau_181_/Aβ_42_ ratio. Fifth, cognitive changes were analyzed using linear mixed-effects models (LMMs) instead of GLMs, modeling longitudinal cognitive trajectories with interaction terms (Time × SRP-max ROI and Time × SUVR-max ROI) and adjusting for age, sex, education, and baseline cognitive scores. Sixth, cognitive fluctuation was quantified as intraindividual variability following a previous LMM-based approach.^33^ Domain-specific LMMs were fitted for each participant, adjusting for age, sex, education, and baseline scores; subject-specific residuals were extracted, and their standard deviation represented intraindividual variability. These values were then used as outcomes in GLMs to assess associations with regional brain metabolism. Seventh, we compared model fits across different combinations using LMMs, evaluating whether adding SRP-max ROI or SUVR-max ROI improved prediction of longitudinal cognitive trajectories beyond SAA positivity and AD signature metabolism.

**Supplementary results**

*Sensitivity analyses*

We additionally compared brain metabolism between NC and MCI participants, as well as between NC and dementia participants (**Supplementary Figure 4**). In the SRP analyses (**Supplementary Figure 4 A-1 to A-3**), compared with the NC group, both MCI^SAA+^ and MCI^SAA−^ groups exhibited hypermetabolism in the pons, cerebellum, midbrain, basal ganglia, insular, anterior cingulate, and primary motor cortex, along with hypometabolism in the medial temporal, inferior temporal, and parietal cortices. The MCI^SAA+^ group, compared with the NC group, additionally showed hypermetabolism in the olfactory cortex, whereas the MCI^SAA−^ group exhibited hypermetabolism in the occipital cortex. The MCI^SAA+^ group exhibited increased metabolism in the cerebellum, olfactory cortex, thalamus, caudate, right putamen, insula, and cingulate cortex, along with hypometabolism in the temporoparietal cortex compared with the MCI^SAA−^ group at an uncorrected threshold (p < 0.05). However, these differences did not survive corrections for multiple comparisons (**Supplementary Figure 4 A-3**). In the SUVR analyses (**Supplementary Figure 4 B-1 to B-3**), both the MCI^SAA+^ and MCI^SAA−^ groups showed widespread hypometabolism compared with the NC group, with the MCI^SAA+^ group additionally showing occipital hypometabolism. The MCI^SAA+^ group showed hypometabolism in the temporo-parieto-occipital cortices compared with the MCI^SAA−^ group at an uncorrected threshold (p < 0.05). However, these differences did not survive corrections for multiple comparisons (**Supplementary Figure 4 B-3**). In the comparison between NC and dementia groups (**Supplementary Figure 4 C-1 to D-3**), both Dementia^SAA+^ and Dementia^SAA−^ groups showed similar but more pronounced metabolic alterations compared with the NC group, regardless of whether assessed using SRP or SUVR methods. The Dementia^SAA+^ group exhibited increased metabolism in the cerebellum, olfactory cortex, putamen, insula, cingulate cortex, and primary motor cortex, along with hypometabolism in the occipital and dorsolateral prefrontal cortex compared with the Dementia^SAA−^ group when assessed using the SRP method at an uncorrected threshold (p < 0.05). However, these differences did not survive corrections for multiple comparisons (**Supplementary Figure 4 C-3**). Using the SUVR method, the Dementia^SAA+^ group showed hypometabolism in the temporo-parieto-occipital and dorsolateral prefrontal cortices compared with the Dementia^SAA−^ group However, these differences did not survive corrections for multiple comparisons at an uncorrected threshold (p < 0.05). (**Supplementary Figure 4 D-3**). The AD^SAA+^ group consistently demonstrated a trend toward increased putaminal SRP and decreased occipital SUVR compared with the AD^SAA−^ group in both the MCI and dementia stages.

In the GLM assessing the independent effects of disease stage (MCI vs. dementia) and SAA positivity on FDG-SRP among AD participants, the AD^SAA+^ group showed increased metabolism in the bilateral putamen, orbitofrontal cortex, anterior cingulate cortex, and cerebellum, and decreased metabolism in the occipitoparietal cortex compared with the AD^SAA−^ group (**Supplementary** **Figure 6 A-1**). Compared with the MCI stage, the dementia stage was associated with increased metabolism in the cerebellum, pons, medial occipital cortices, thalamus, basal ganglia, orbitofrontal cortices, insula, medial frontal cortices, and sensorimotor cortices, with particular emphasis on the vermis and putamen, while showing decreased metabolism in the temporoparietal cortices and dorsolateral frontal cortices (**Supplementary** **Figure 6 A-2**). GLMs for FDG-SUVR showed that the AD^SAA+^ group exhibited decreased metabolism in the bilateral occipital cortices compared to the AD^SAA−^ group (**Supplementary** **Figure 6 B-1**). Compared with the MCI stage, the dementia stage exhibited increased metabolism in the vermis, while showing decreased metabolism across the entire cortex except for the sensorimotor cortex, the calcarine fissure and surrounding cortex, and the putamen (**Supplementary** **Figure 6 F-2**). Among these regions with reduced metabolism, the parietal cortices were most prominently affected.

Sensitivity analyses of the independent effects of right putamen SRP and left middle occipital gyrus SUVR on cognition, stratified by MCI and dementia stages, showed that right putaminal hypermetabolism was the only metabolic alteration that predicted global cognitive worsening: ADAS worsening in MCI^SAA+^ and MMSE decline in both Dementia^SAA+^ and Dementia^SAA−^ groups, although the latter association did not remain statistically significant after correction for multiple comparisons in the Dementia^SAA+^ group (**Supplementary Table 3**). In contrast, hypometabolism in the left middle occipital gyrus was associated with faster decline in executive function in the MCI^SAA−^ group. Sensitivity analyses of cognitive variability showed that hypometabolism in the left middle occipital gyrus was associated with higher variability in MMSE, ADAS, memory, language, and executive function in the Dementia^SAA−^ group, and with higher variability in ADAS in the MCI^SAA+^ group, while hypermetabolism in the right putamen was associated with higher variability in MMSE in the Dementia^SAA+^ group, although the latter association did not remain statistically significant after correction for multiple comparison.

Sensitivity analyses of the associations among SAA positivity, right putamen SRP, and AD signature metabolism with cognition, stratified by MCI and dementia stages, showed that right putamen SRP consistently improved model fit for global cognitive decline across both stages, independent of SAA positivity and AD signature metabolism (**Supplementary Tables 4–5**). In contrast, left middle occipital hypometabolism did not improve model fit for global cognitive decline in either MCI or dementia (**Supplementary Tables 6–7**).

Sensitivity analyses using amyloid PET-based CL values instead of the log-transformed CSF pTau_181_/Aβ_42_ ratio yielded results consistent with the main findings (**Supplementary Table 8** and **Supplementary Figure 5**). Sensitivity analyses of longitudinal cognitive trajectories using LMMs are presented in **Supplementary Table 9**. In the NC group, alterations in right putamen SRP or left middle occipital gyrus SUVR were not associated with longitudinal decline in any cognitive scores. In the AD^SAA−^ group, higher right putamen SRP and lower left middle occipital gyrus SUVR were both associated with greater longitudinal worsening in MMSE and ADAS scores in multivariable models. Additionally, lower middle occipital gyrus SUVR was associated with greater longitudinal decline in memory, language, visuospatial, and executive scores. In the AD^SAA+^ group, multivariable models revealed that higher right putaminal SRP was independently associated with greater longitudinal worsening in MMSE and ADAS scores, while left middle occipital SUVR was not. The sensitivity analysis using LMM-derived cognitive variability yielded results largely consistent with the main findings (**Supplementary Table 10**).

The sensitivity analyses using LLMs to evaluate whether adding right putamen SRP or left middle occipital gyrus SUVR provided additional predictive value beyond SAA positivity and AD hypometabolism showed results consistent with the GLM-based analyses (GLM-based: **Table 4** and **Supplementary Table 2**; LLM-based: **Supplementary Tables 11–12**), confirming that right putamen SRP consistently demonstrated additive predictive value, whereas left middle occipital gyrus SUVR did not.

**Supplementary Figure 1.** Flowchart of study participants


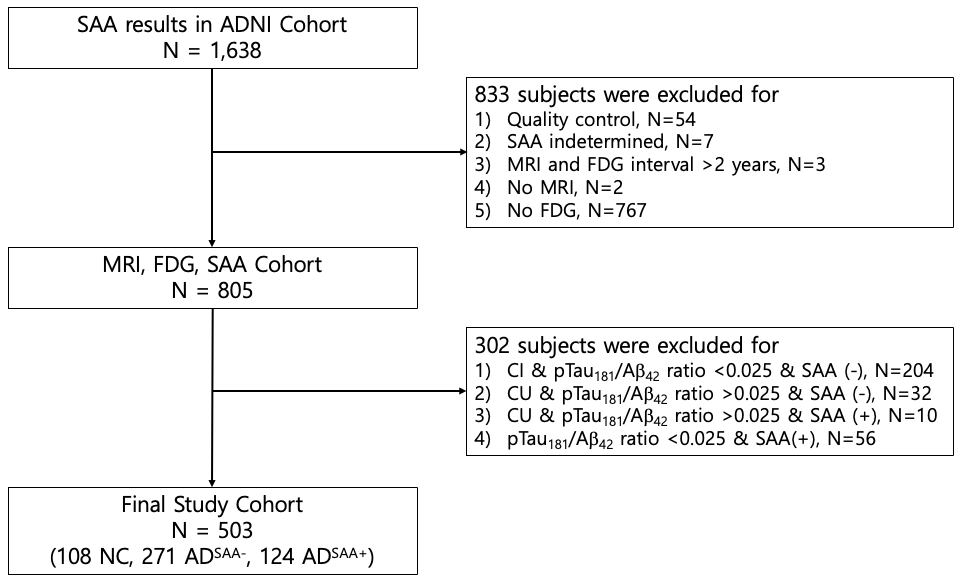


Abbreviations: ADNI, Alzheimer’s Disease Neuroimaging Initiative; CI, cognitively impaired; CU, cognitively unimpaired; FDG, fluorodeoxyglucose; NC, normal controls; MRI, magnetic resonance imaging; SAA, seed amplification assays.

**Supplementary Figure 2.** AD signature brain metabolism


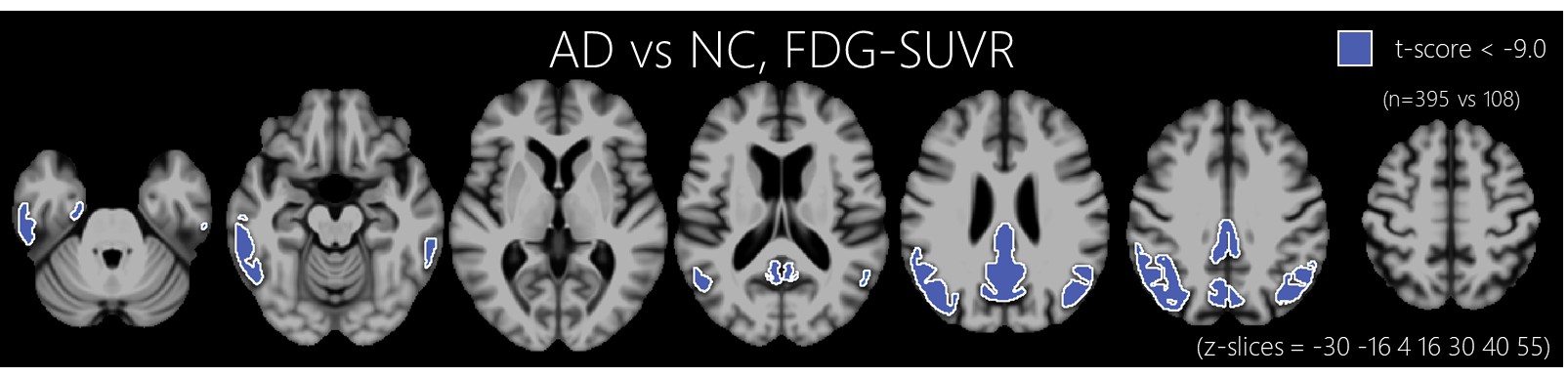


The regions shown were identified by voxel-wise general linear models on FDG-SUVR comparing individuals with Alzheimer’s disease (AD) and normal controls (NCs), thresholded at t = –9, after controlling for age, sex, and education.

**Supplementary Figure 3.** Comparison of regional brain metabolism using SRP and SUVR between AD^SAA−^ and AD^SAA+^ groups


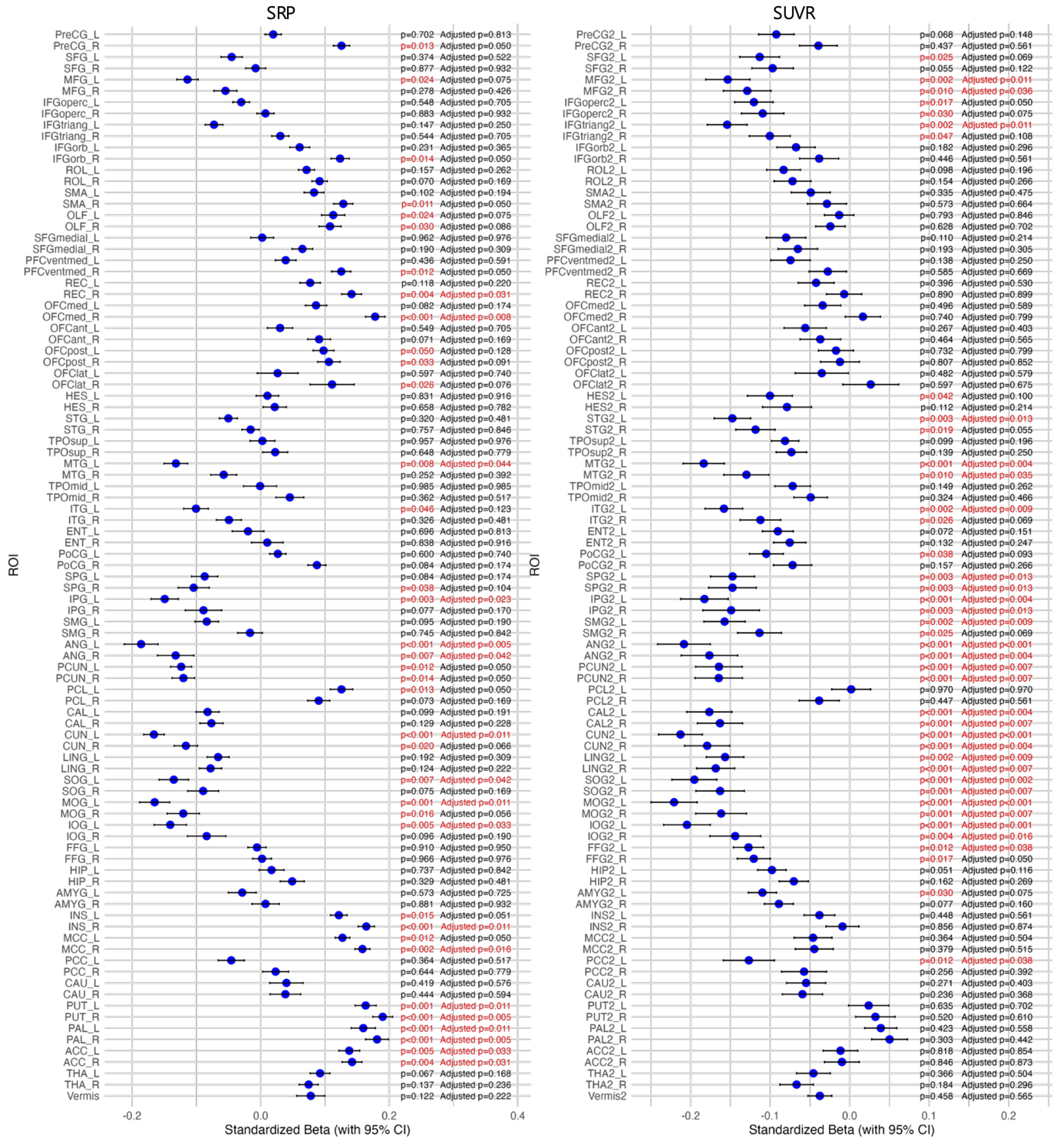


This figure presents a comparison of regional brain metabolism between AD^SAA−^ and AD^SAA+^ groups, analyzed using SRP and SUVR imaging methods. The results are based on the general linear models for regional metabolism after controlling for age, sex, and education. Abbreviations with "_L" indicate the left hemisphere, and abbreviations with "_R" indicate the right hemisphere. Abbreviations: ACC, anterior cingulate cortex; AD, Alzheimer’s disease; AMYG, amygdala; ANG, angular gyrus; CAL, calcarine fissure and surrounding cortex; BFV, basal forebrain volume; CAU, caudate nucleus; CUN, cuneus; DAT-PC, dopamine transporter uptake in the posterior caudate; ENT, entorhinal cortex; FFG, fusiform gyrus; HES, Heschl’s gyrus; HIP, hippocampus; IFGorb, IFG pars orbitalis; IFGoperc, inferior frontal gyrus, opercular part; IFGtriang, inferior frontal gyrus, triangular part; INS, insula; IOG, inferior occipital gyrus; IPG, inferior parietal gyrus, excluding supramarginal; ITG, inferior temporal gyrus; LING, lingual gyrus; MCC, middle cingulate & paracingulate gyrus; MFG, middle frontal gyrus; MOG, middle occipital gyrus; MTG, middle temporal gyrus; OLF, olfactory cortex; OFCant, anterior orbital gyrus; OFCmed, medial orbital gyrus; OFCpost, posterior orbital gyrus; OFClat, lateral orbital gyrus; PAL, pallidum; PCC, posterior cingulate gyrus; PCUN, precuneus; PFCventmed, superior frontal gyrus, medial orbital; PCL, paracentral lobule; PoCG, postcentral gyrus; PreCG, precentral gyrus; PUT, putamen; REC, gyrus rectus; ROL, rolandic operculum; SMA, supplementary motor area; SMG, supramarginal gyrus; SFG, superior frontal gyrus; SFGmedial, superior frontal gyrus, medial; SOG, superior occipital gyrus; SPG, superior parietal gyrus; STG, superior temporal gyrus; THA, thalamus; TPOmid, temporal pole-middle temporal gyrus; TPOsup, temporal pole-superior temporal gyrus.

**Supplementary Figure 4.** Group comparisons of brain metabolism across disease stages and SAA status

**
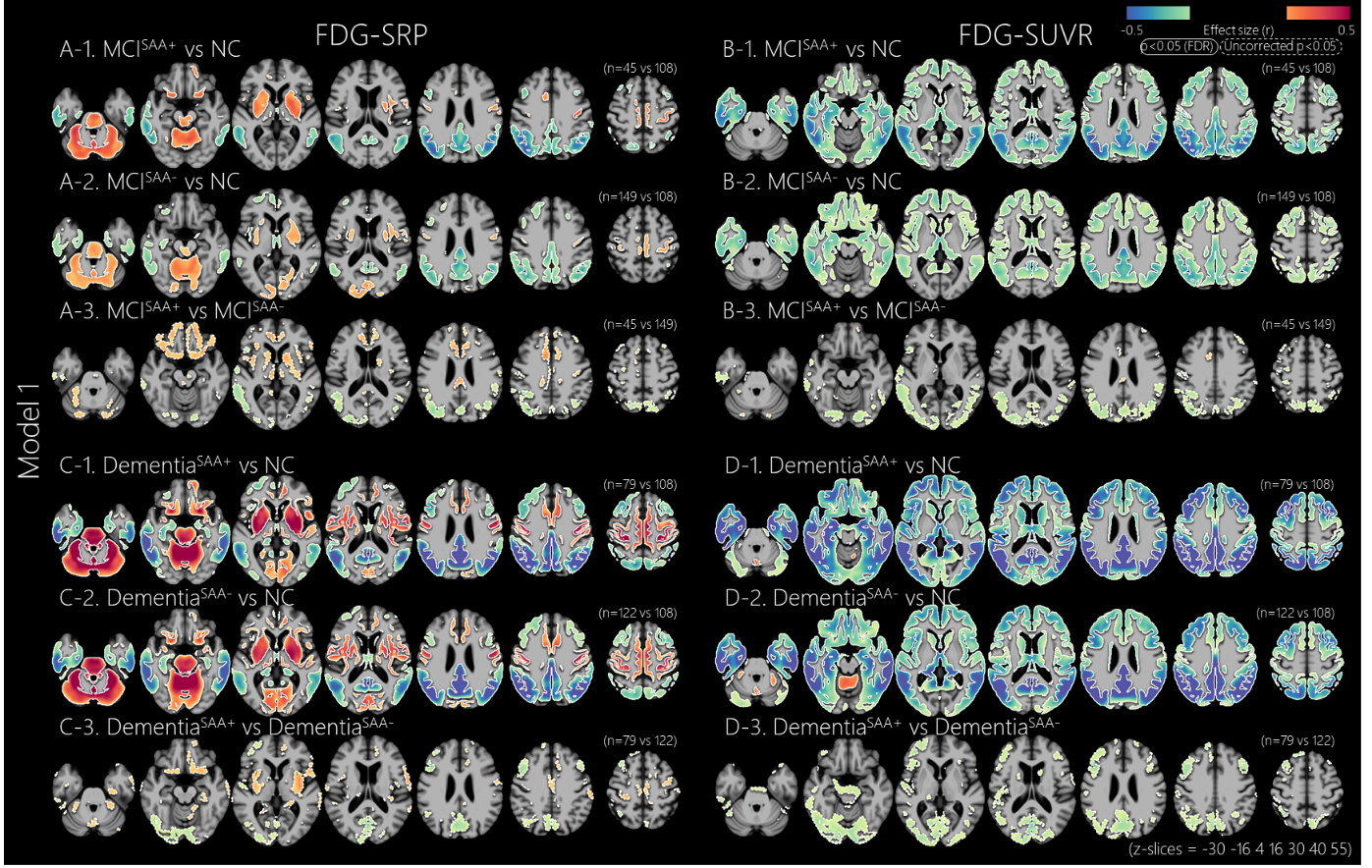
**

Voxel-wise analyses of FDG metabolism using general linear models are shown, adjusted for age, sex, and education. Red areas show higher metabolism in the first group, and blue areas show lower metabolism. Solid lines indicate significant regions that survived correction for false discovery rate (FDR) multiple comparisons (corrected p< 0.05, A-1, A-2, B-1, B-2, C-1, C-2, D-1, D-2), whereas dashed lines indicate significant regions at the uncorrected threshold (uncorrected p < 0.05, A-3, B-3, C-3, D-3).

**Supplementary Figure 5.** Interaction of SAA positivity and amyloid PET centiloids on left middle occipital gyrus SUVR in AD participants


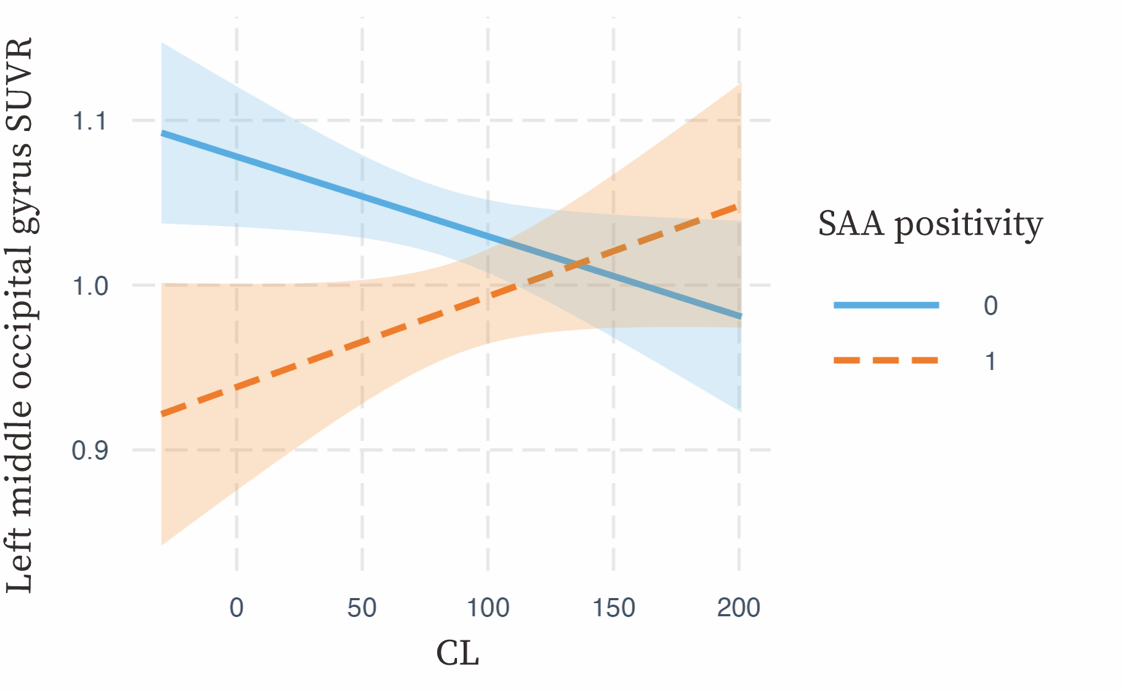


The results are based on the general linear models for left middle occipital SUVR using amyloid CL as a predator after controlling for age, sex, education, and baseline MMSE score, separately performed in SAA-positive and SAA-negative AD participants. Abbreviations: AD, Alzheimer’s disease; CL, centiloids; SAA, seed amplification assays; SUVR, standardized uptake value ratio.

**Supplementary Figure 6.** Independent effects of disease stage and SAA positivity on brain metabolism in participants with AD

**
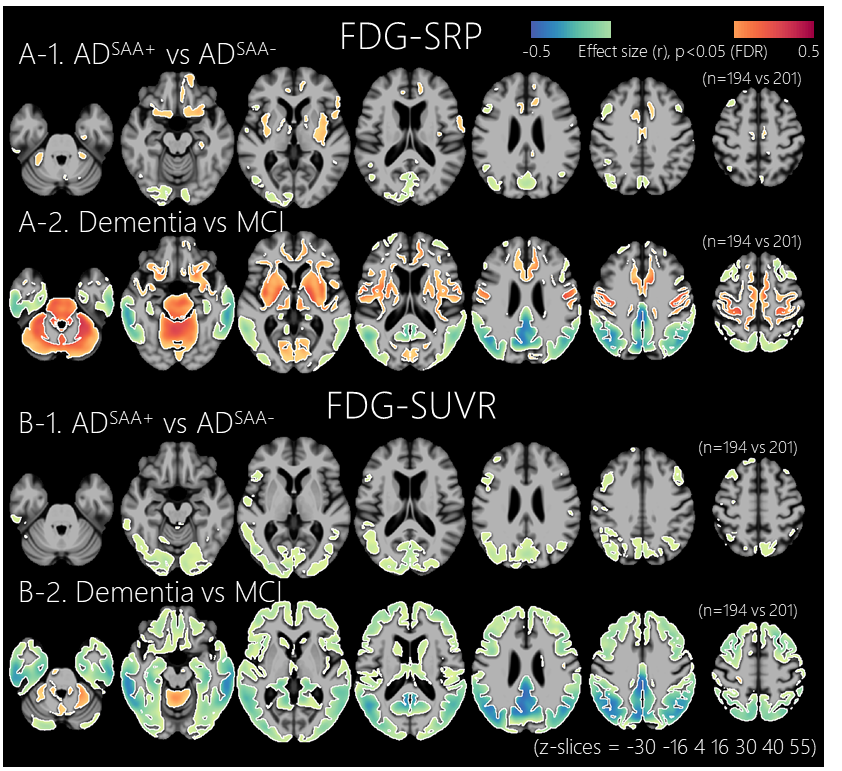
**

Voxel-wise analyses of FDG metabolism were conducted using general linear models with SAA positivity and disease stage (MCI vs. Dementia) as independent predictors, adjusted for age, sex, and education. Red areas show higher metabolism in the first group, and blue areas show lower metabolism. Displayed results are thresholded at a false discovery rate–corrected significance level of p < 0.05.

**Supplementary Figure 7.** Associations between mean cerebellar gray matter SRP and voxel-wise SRP metabolism in participants with AD


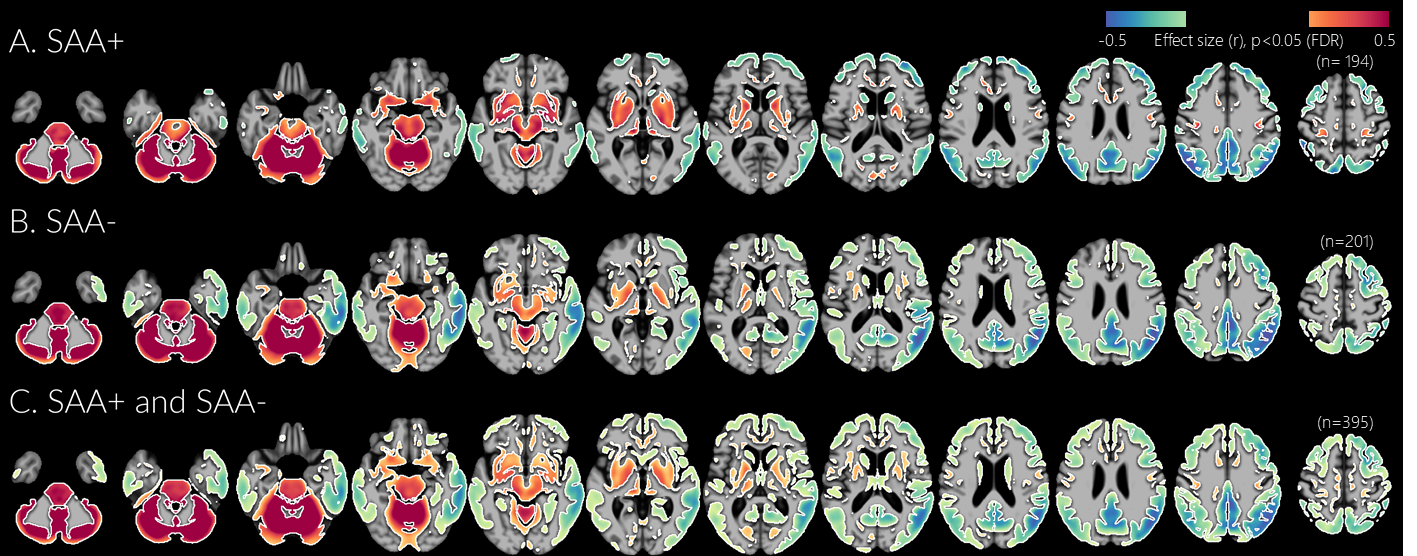


The results are based on the general linear models for regional brain metabolism using mean cerebellar gray matter metabolism as a predictor after controlling for age, sex, and education. Red areas show positive correlations, and blue areas show negative correlations. Displayed results are thresholded at a false discovery rate–corrected significance level of p < 0.05.

**Supplementary Table 1.** Associations of left middle occipital gyrus SUVR with right putamen SRP

| Predictors | Lt MOG SUVR | | Lt MOG SUVR × SAA positivity | | Lt MOG SUVR ×  log- pTau_181_/Aβ_42_ ratio | |
| --- | --- | --- | --- | --- | --- | --- |
| Subjects | B | P | B | P | B | P |
| NC | -0.05 | 0.553 | - | - | - | - |
| AD^SAA−^ | -0.15 | **0.018** | - | - | - | - |
| AD^SAA+^ | -0.35 | **<0.001** | - | - | - | - |
| AD participants | -0.24 | **<0.001** | - | - | - | - |
| AD participants | -0.16 | **0.007** | -0.79 | **0.023** | - | - |
| AD participants | -0.57 | 0.080 | - | - | -0.52 | 0.302 |
| Whole participants | -0.24 | **<0.001** | - | - | - | - |
| Whole participants | -0.17 | **<0.001** | -0.67 | **0.025** | - | - |
| Whole participants | -0.48 | **0.013** | - | - | -0.60 | 0.176 |

The results are based on the general linear models for right putamen SRP after controlling for age, sex, education, and baseline MMSE score. Abbreviations: AD, Alzheimer’s disease; Lt MOG SUVR, left middle occipital gyrus standardized uptake value ratio; NC, normal control; log-pTau_181_/Aβ_42_ ratio, log-transformed phosphorylated tau/Aβ42 ratio; SAA, seeding amplification assays.

**Supplementary Table 2**. Effects of SAA positivity, left middle occipital gyrus SUVR, and AD signature metabolism on rate/variability of cognitive changes in AD participants

| Predictors | SAA positivity | | Lt MOG SUVR | | AD signature metabolism | |  |  |  |
| --- | --- | --- | --- | --- | --- | --- | --- | --- | --- |
| Outcomes | B | P | B | P | B | P | AIC | P^1 vs. 2^ | P^3 vs. 4^ |
| **GLM 1** |  |  |  |  |  |  |  |  |  |
| *Annual change* |  |  |  |  |  |  |  |  |  |
| MMSE | -0.13 | **0.013** | - | - | - | - | 1686.69 | **<0.001** | - |
| ADAS | 0.16 | **0.002** | - | - | - | - | 2328.09 | **0.001** | - |
| Memory | -0.22 | **<0.001** | - | - | - | - | 360.22 | **0.004** | - |
| Language | -0.17 | **0.001** | - | - | - | - | 595.60 | **0.010** | - |
| Visuospatial | -0.13 | **0.010** | - | - | - | - | 836.77 | **0.011** | - |
| Executive | -0.11 | 0.035 | - | - | - | - | 490.34 | **<0.001** | - |
| *Variability* |  |  |  |  |  |  |  |  |  |
| MMSE | 0.14 | **0.010** | - | - | - | - | 1352.10 | **<0.001** | - |
| ADAS | 0.11 | 0.034 | - | - | - | - | 1937.54 | **<0.001** | - |
| Memory | 0.09 | 0.096 | - | - | - | - | 29.13 | **<0.001** | - |
| Language | 0.07 | 0.214 | - | - | - | - | 116.26 | **0.001** | - |
| Visuospatial | 0.10 | 0.058 | - | - | - | - | 192.69 | **0.001** | - |
| Executive | 0.04 | 0.407 | - | - | - | - | 35.96 | **<0.001** | - |
| **GLM 2** |  |  |  |  |  |  |  |  |  |
| *Annual change* |  |  |  |  |  |  |  |  |  |
| MMSE | -0.09 | 0.079 | 0.20 | **<0.001** | - | - | 1673.15 | **<0.001** | - |
| ADAS | 0.13 | **0.011** | -0.17 | **0.001** | - | - | 2319.80 | **0.001** | - |
| Memory | -0.19 | **<0.001** | 0.16 | **0.004** | - | - | 353.59 | **0.004** | - |
| Language | -0.14 | **0.008** | 0.14 | **0.010** | - | - | 590.90 | **0.010** | - |
| Visuospatial | -0.11 | 0.046 | 0.14 | **0.011** | - | - | 832.11 | **0.011** | - |
| Executive | -0.08 | 0.129 | 0.21 | **<0.001** | - | - | 478.10 | **<0.001** | - |
| *Variability* |  |  |  |  |  |  |  |  |  |
| MMSE | 0.09 | 0.103 | -0.28 | **<0.001** | - | - | 1328.27 | **<0.001** | - |
| ADAS | 0.07 | 0.200 | -0.27 | **<0.001** | - | - | 1914.90 | **<0.001** | - |
| Memory | 0.05 | 0.338 | -0.20 | **<0.001** | - | - | 17.58 | **<0.001** | - |
| Language | 0.03 | 0.564 | -0.19 | **0.001** | - | - | 106.54 | **0.001** | - |
| Visuospatial | 0.06 | 0.233 | -0.18 | **0.001** | - | - | 184.16 | **0.001** | - |
| Executive | 0.01 | 0.876 | -0.24 | **<0.001** | - | - | 19.56 | **<0.001** | - |
| **GLM 3** |  |  |  |  |  |  |  |  |  |
| *Annual change* |  |  |  |  |  |  |  |  |  |
| MMSE | -0.08 | 0.098 | - | - | 0.35 | **<0.001** | 1646.00 | - | 0.305 |
| ADAS | 0.12 | 0.014 | - | - | -0.31 | **<0.001** | 2300.68 | - | 0.347 |
| Memory | -0.18 | **<0.001** | - | - | 0.27 | **<0.001** | 339.88 | - | 0.526 |
| Language | -0.13 | 0.011 | - | - | 0.24 | **<0.001** | 579.39 | - | 0.529 |
| Visuospatial | -0.09 | 0.087 | - | - | 0.26 | **<0.001** | 816.59 | - | 0.255 |
| Executive | -0.07 | 0.189 | - | - | 0.32 | **<0.001** | 461.93 |  | 0.849 |
| *Variability* |  |  |  |  |  |  |  |  |  |
| MMSE | 0.08 | 0.099 | - | - | -0.39 | **<0.001** | 1305.17 | - | 0.831 |
| ADAS | 0.06 | 0.237 | - | - | -0.44 | **<0.001** | 1880.17 | - | 0.499 |
| Memory | 0.04 | 0.410 | - | - | -0.32 | **<0.001** | -0.49 | - | 0.638 |
| Language | 0.03 | 0.605 | - | - | -0.26 | **<0.001** | 97.70 | - | 0.771 |
| Visuospatial | 0.07 | 0.175 | - | - | -0.15 | **0.006** | 187.01 | - | 0.074 |
| Executive | 0.002 | 0.974 | - | - | -0.32 | **<0.001** | 9.70 | - | 0.451 |
| **GLM 4** |  |  |  |  |  |  |  |  |  |
| *Annual change* |  |  |  |  |  |  |  |  |  |
| MMSE | -0.09 | 0.078 | -0.07 | 0.305 | 0.41 | **<0.001** | 1646.92 | - | 0.305 |
| ADAS | 0.13 | **0.011** | 0.07 | 0.347 | -0.36 | **<0.001** | 2301.77 | - | 0.347 |
| Memory | -0.18 | **<0.001** | -0.05 | 0.526 | 0.30 | **<0.001** | 341.47 | - | 0.526 |
| Language | -0.13 | **0.010** | -0.05 | 0.529 | 0.28 | **0.001** | 580.99 | - | 0.529 |
| Visuospatial | -0.09 | 0.068 | -0.09 | 0.255 | 0.32 | **<0.001** | 817.26 | - | 0.255 |
| Executive | -0.07 | 0.186 | -0.02 | 0.849 | 0.34 | **<0.001** | 463.89 | - | 0.849 |
| *Variability* |  |  |  |  |  |  |  |  |  |
| MMSE | 0.08 | 0.107 | -0.02 | 0.831 | -0.38 | **<0.001** | 1307.12 | - | 0.831 |
| ADAS | 0.06 | 0.212 | 0.05 | 0.499 | -0.48 | **<0.001** | 1881.70 | - | 0.499 |
| Memory | 0.04 | 0.383 | 0.04 | 0.638 | -0.35 | **<0.001** | 1.29 | - | 0.638 |
| Language | 0.03 | 0.630 | -0.02 | 0.771 | -0.24 | **0.003** | 99.61 | - | 0.771 |
| Visuospatial | 0.06 | 0.247 | -0.14 | 0.074 | -0.05 | 0.529 | 185.75 | - | 0.074 |
| Executive | 0.00 | 0.983 | -0.06 | 0.451 | -0.27 | **0.001** | 11.12 | - | 0.451 |

The results are based on general linear model for the rate/variability of cognitive changes. GLM 1 included a SAA positivity; GLM 2 included SAA positivity and left MOG SUVR; GLM 3 included SAA positivity and AD signature metabolism; and GLM 4 included all three predictors. All models were adjusted for baseline cognitive performance, age, sex, and education. P values for model comparison were obtained from likelihood ratio tests comparing GLM 1 vs. GLM 2 (P^1 vs. 2^) and GLM 3 vs. GLM 4 (P^3 vs. 4^), respectively. Multiple comparisons were corrected using the false discovery rate (FDR) across 12 cognitive outcomes. The p-values reported are uncorrected, and those that remained significant after FDR correction are shown in bold. Abbreviations: AD, Alzheimer’s disease; ADAS, Alzheimer’s disease assessment scale; AIC, Akaike information criterion; B, standardized beta coefficient; FDR, false discovery rate; Lt MOG SUVR, left middle occipital gyrus metabolism assessed by standardized uptake value ratio; MMSE, mini-mental state examination; SAA, seed amplification assays.

**Supplementary Table 3.** Association between right putamen SRP or left middle occipital gyrus SUVR and rate/variability of cognitive changes

|  | MCI^SAA−^ (N = 149) | | | | Dementia^SAA−^ (N = 122) | | | | MCI^SAA+^ (N = 45) | | | | Dementia^SAA+^ (N = 79) | | | |
| --- | --- | --- | --- | --- | --- | --- | --- | --- | --- | --- | --- | --- | --- | --- | --- | --- |
|  | Rt PUT SRP | | Lt MOG SUVR | | Rt PUT SRP | | Lt MOG SUVR | | Rt PUT SRP | | Lt MOG SUVR | | Rt PUT SRP | | Lt MOG SUVR | |
|  | B | P | B | P | B | P | B | P | B | P | B | P | B | P | B | P |
| **Univariable model** |  |  |  |  |  |  |  |  |  |  |  |  |  |  |  |  |
| *Annual change* |  |  |  |  |  |  |  |  |  |  |  |  |  |  |  |  |
| MMSE | -0.12 | 0.188 | 0.16 | 0.059 | -0.36 | **<0.001** | 0.27 | **0.004** | -0.30 | 0.100 | 0.39 | **0.011** | -0.25 | 0.028 | 0.02 | 0.854 |
| ADAS | 0.14 | 0.132 | -0.13 | 0.160 | 0.20 | 0.044 | -0.16 | 0.083 | 0.58 | **<0.001** | -0.37 | **0.016** | 0.26 | 0.019 | -0.16 | 0.137 |
| Memory | -0.07 | 0.469 | 0.22 | 0.012 | -0.18 | 0.077 | 0.25 | **0.009** | -0.42 | 0.017 | 0.26 | 0.095 | 0.13 | 0.282 | -0.06 | 0.608 |
| Language | -0.10 | 0.280 | 0.13 | 0.155 | -0.06 | 0.558 | 0.18 | 0.065 | -0.45 | 0.013 | 0.36 | 0.029 | -0.10 | 0.390 | -0.07 | 0.543 |
| Visuospatial | 0.01 | 0.885 | 0.08 | 0.351 | -0.14 | 0.157 | 0.07 | 0.454 | -0.28 | 0.127 | 0.28 | 0.088 | -0.01 | 0.949 | 0.04 | 0.720 |
| Executive | -0.07 | 0.472 | 0.31 | **0.001** | -0.10 | 0.351 | 0.14 | 0.146 | -0.30 | 0.093 | 0.36 | 0.030 | 0.01 | 0.963 | 0.09 | 0.459 |
| *Variability* |  |  |  |  |  |  |  |  |  |  |  |  |  |  |  |  |
| MMSE | 0.07 | 0.427 | -0.07 | 0.419 | 0.29 | **0.003** | -0.41 | **<0.001** | 0.36 | 0.037 | -0.37 | **0.016** | 0.29 | 0.013 | -0.16 | 0.178 |
| ADAS | 0.17 | 0.063 | -0.02 | 0.809 | 0.25 | **0.010** | -0.31 | **0.001** | 0.34 | 0.057 | -0.50 | **0.001** | 0.31 | 0.007 | -0.33 | **0.005** |
| Memory | 0.12 | 0.210 | -0.10 | 0.244 | 0.25 | **0.015** | -0.32 | **0.001** | 0.43 | 0.011 | -0.38 | **0.009** | 0.04 | 0.761 | -0.09 | 0.458 |
| Language | 0.08 | 0.403 | -0.04 | 0.662 | 0.15 | 0.163 | -0.32 | **0.001** | 0.33 | 0.052 | -0.28 | 0.065 | 0.18 | 0.126 | -0.11 | 0.374 |
| Visuospatial | 0.03 | 0.771 | 0.00 | 0.992 | 0.24 | **0.016** | -0.23 | **0.017** | 0.26 | 0.149 | -0.16 | 0.337 | 0.13 | 0.293 | -0.34 | **0.005** |
| Executive | 0.03 | 0.707 | -0.15 | 0.111 | 0.13 | 0.218 | -0.36 | **<0.001** | 0.12 | 0.516 | -0.22 | 0.191 | 0.10 | 0.448 | -0.18 | 0.166 |
| **Multivariable model** |  |  |  |  |  |  |  |  |  |  |  |  |  |  |  |  |
| *Annual change* |  |  |  |  |  |  |  |  |  |  |  |  |  |  |  |  |
| MMSE | -0.10 | 0.273 | 0.15 | 0.082 | -0.30 | **0.003** | 0.18 | 0.060 | -0.16 | 0.363 | 0.34 | 0.035 | -0.28 | 0.022 | -0.09 | 0.448 |
| ADAS | 0.12 | 0.184 | -0.11 | 0.224 | 0.16 | 0.117 | -0.12 | 0.235 | 0.50 | **0.003** | -0.22 | 0.120 | 0.23 | 0.055 | -0.07 | 0.521 |
| Memory | -0.04 | 0.682 | 0.21 | 0.015 | -0.11 | 0.283 | 0.22 | 0.029 | -0.36 | 0.048 | 0.16 | 0.294 | 0.12 | 0.346 | -0.01 | 0.935 |
| Language | -0.09 | 0.353 | 0.12 | 0.191 | -0.01 | 0.955 | 0.18 | 0.082 | -0.37 | 0.040 | 0.27 | 0.096 | -0.15 | 0.237 | -0.13 | 0.308 |
| Visuospatial | 0.03 | 0.780 | 0.08 | 0.337 | -0.13 | 0.215 | 0.03 | 0.745 | -0.21 | 0.268 | 0.23 | 0.179 | 0.01 | 0.952 | 0.05 | 0.722 |
| Executive | -0.02 | 0.828 | 0.30 | **0.001** | -0.06 | 0.562 | 0.13 | 0.210 | -0.21 | 0.226 | 0.30 | 0.070 | 0.04 | 0.743 | 0.11 | 0.422 |
| *Variability* |  |  |  |  |  |  |  |  |  |  |  |  |  |  |  |  |
| MMSE | 0.07 | 0.490 | -0.06 | 0.481 | 0.18 | 0.069 | -0.35 | **<0.001** | 0.23 | 0.192 | -0.29 | 0.077 | 0.27 | 0.035 | -0.05 | 0.676 |
| ADAS | 0.17 | 0.067 | 0.001 | 0.991 | 0.17 | 0.092 | -0.27 | **0.005** | 0.16 | 0.323 | -0.46 | **0.003** | 0.22 | 0.070 | -0.24 | 0.051 |
| Memory | 0.10 | 0.266 | -0.09 | 0.312 | 0.16 | 0.122 | -0.28 | **0.004** | 0.33 | 0.052 | -0.30 | 0.043 | 0.0002 | 0.999 | -0.09 | 0.501 |
| Language | 0.07 | 0.430 | -0.03 | 0.731 | 0.05 | 0.623 | -0.31 | **0.002** | 0.27 | 0.125 | -0.22 | 0.158 | 0.17 | 0.200 | -0.04 | 0.743 |
| Visuospatial | 0.03 | 0.770 | 0.003 | 0.975 | 0.18 | 0.076 | -0.17 | 0.081 | 0.23 | 0.226 | -0.10 | 0.559 | 0.02 | 0.891 | -0.34 | 0.009 |
| Executive | 0.01 | 0.880 | -0.14 | 0.121 | 0.04 | 0.734 | -0.35 | **0.001** | 0.06 | 0.747 | -0.21 | 0.244 | 0.04 | 0.769 | -0.16 | 0.235 |

The results are based on general linear models using right putamen SRP or left middle occipital gyrus SUVR as predictors. The univariable model included either right putamen SRP or left middle occipital gyrus SUVR as a predictor, whereas the multivariable model includes both as predictors. Covariates include age, sex, education, and baseline cognitive scores (e.g., baseline MMSE, baseline ADAS). Multiple comparisons were corrected using the FDR method across 12 cognitive outcomes (6 annual change outcomes + 6 variability outcomes) within each group. The p values reported are uncorrected, and those that remained significant after FDR correction are shown in bold. Abbreviations: AD, Alzheimer’s disease; ADAS, Alzheimer’s disease assessment scale; B, standardized beta coefficient; FDR, false discovery rate; Lt MOG SUVR, left middle occipital gyrus metabolism assessed by standardized uptake value ratio; MCI, mild cognitive impairment; MMSE, mini-mental state examination; NC, normal control; Rt PUT SRP, right putaminal metabolism assessed by subject residual profile method; SAA, seed amplification assays.

**Supplementary Table 4.** Effects of SAA positivity, right putamen SRP, and AD signature metabolism on the rate/variability of cognitive changes in MCI due to AD participants

| Predictors | SAA positivity | | Rt PUT SRP | | AD signature metabolism | |  |  |  |
| --- | --- | --- | --- | --- | --- | --- | --- | --- | --- |
| Outcomes | B | P | B | P | B | P | AIC | P^1 vs. 2^ | P^3 vs. 4^ |
| **GLM 1** |  |  |  |  |  |  |  |  |  |
| *Annual change* |  |  |  |  |  |  |  |  |  |
| MMSE | -0.10 | 0.191 | - | - | - | - | 769.97 | 0.046 | - |
| ADAS | 0.20 | **0.008** | - | - | - | - | 1062.51 | **0.002** | - |
| Memory | -0.24 | **0.001** | - | - | - | - | 110.68 | 0.045 | - |
| Language | -0.11 | 0.137 | - | - | - | - | 246.09 | 0.028 | - |
| Visuospatial | -0.13 | 0.085 | - | - | - | - | 269.50 | 0.349 | - |
| Executive | -0.06 | 0.430 | - | - | - | - | 183.51 | 0.087 | - |
| *Variability* |  |  |  |  |  |  |  |  |  |
| MMSE | 0.16 | 0.034 | - | - | - | - | 659.29 | 0.047 | - |
| ADAS | 0.12 | 0.108 | - | - | - | - | 953.63 | **0.006** | - |
| Memory | 0.06 | 0.438 | - | - | - | - | 12.98 | 0.017 | - |
| Language | 0.08 | 0.270 | - | - | - | - | 54.77 | 0.054 | - |
| Visuospatial | 0.15 | 0.040 | - | - | - | - | 58.98 | 0.426 | - |
| Executive | 0.01 | 0.910 | - | - | - | - | 32.64 | 0.481 | - |
| **GLM 2** |  |  |  |  |  |  |  |  |  |
| *Annual change* |  |  |  |  |  |  |  |  |  |
| MMSE | -0.08 | 0.296 | -0.16 | 0.046 | - | - | 767.82 | 0.046 | - |
| ADAS | 0.17 | 0.020 | 0.25 | **0.002** | - | - | 1054.46 | **0.002** | - |
| Memory | -0.22 | **0.002** | -0.16 | 0.045 | - | - | 108.48 | 0.045 | - |
| Language | -0.10 | 0.207 | -0.18 | 0.028 | - | - | 243.04 | 0.028 | - |
| Visuospatial | -0.12 | 0.112 | -0.07 | 0.349 | - | - | 270.59 | 0.349 | - |
| Executive | -0.04 | 0.595 | -0.14 | 0.087 | - | - | 182.45 | 0.087 | - |
| *Variability* |  |  |  |  |  |  |  |  |  |
| MMSE | 0.14 | 0.060 | 0.16 | 0.047 | - | - | 657.17 | 0.047 | - |
| ADAS | 0.10 | 0.200 | 0.22 | **0.006** | - | - | 947.60 | **0.006** | - |
| Memory | 0.04 | 0.632 | 0.19 | 0.017 | - | - | 9.08 | 0.017 | - |
| Language | 0.07 | 0.368 | 0.15 | 0.054 | - | - | 52.90 | 0.054 | - |
| Visuospatial | 0.14 | 0.053 | 0.06 | 0.426 | - | - | 60.32 | 0.426 | - |
| Executive | 0.001 | 0.992 | 0.06 | 0.481 | - | - | 34.12 | 0.481 | - |
| **GLM 3** |  |  |  |  |  |  |  |  |  |
| *Annual change* |  |  |  |  |  |  |  |  |  |
| MMSE | -0.06 | 0.399 | - | - | 0.26 | **<0.001** | 758.96 | - | 0.096 |
| ADAS | 0.17 | 0.019 | - | - | -0.27 | **<0.001** | 1051.36 | - | 0.004 |
| Memory | -0.21 | **0.004** | - | - | 0.29 | **<0.001** | 96.28 | - | 0.086 |
| Language | -0.09 | 0.214 | - | - | 0.19 | **0.017** | 242.14 | - | 0.039 |
| Visuospatial | -0.10 | 0.155 | - | - | 0.17 | **0.020** | 265.90 | - | 0.499 |
| Executive | -0.02 | 0.738 | - | - | 0.33 | **<0.001** | 167.59 |  | 0.189 |
| *Variability* |  |  |  |  |  |  |  |  |  |
| MMSE | 0.12 | 0.103 | - | - | -0.27 | **<0.001** | 647.30 | - | 0.109 |
| ADAS | 0.09 | 0.224 | - | - | -0.31 | **<0.001** | 938.92 | - | 0.012 |
| Memory | 0.02 | 0.785 | - | - | -0.32 | **<0.001** | -3.09 | - | 0.036 |
| Language | 0.06 | 0.412 | - | - | -0.21 | **0.006** | 48.93 | - | 0.076 |
| Visuospatial | 0.14 | 0.056 | - | - | -0.07 | 0.371 | 60.15 | - | 0.488 |
| Executive | -0.02 | 0.771 | - | - | -0.28 | **<0.001** | 21.26 | - | 0.738 |
| **GLM 4** |  |  |  |  |  |  |  |  |  |
| *Annual change* |  |  |  |  |  |  |  |  |  |
| MMSE | -0.05 | 0.516 | -0.13 | 0.096 | 0.25 | **0.001** | 758.05 | - | 0.096 |
| ADAS | 0.15 | 0.040 | 0.22 | 0.004 | -0.25 | **0.001** | 1044.79 | - | 0.004 |
| Memory | -0.19 | 0.007 | -0.13 | 0.086 | 0.28 | **<0.001** | 95.19 | - | 0.086 |
| Language | -0.08 | 0.297 | -0.17 | 0.039 | 0.18 | **0.024** | 239.67 | - | 0.039 |
| Visuospatial | -0.10 | 0.183 | -0.05 | 0.499 | 0.16 | **0.026** | 267.43 | - | 0.499 |
| Executive | -0.01 | 0.877 | -0.10 | 0.189 | 0.32 | **<0.001** | 167.78 | - | 0.189 |
| *Variability* |  |  |  |  |  |  |  |  |  |
| MMSE | 0.11 | 0.141 | 0.13 | 0.109 | -0.26 | **0.001** | 646.61 | - | 0.109 |
| ADAS | 0.07 | 0.345 | 0.19 | 0.012 | -0.29 | **<0.001** | 934.37 | - | 0.012 |
| Memory | 0.00 | 0.973 | 0.16 | 0.036 | -0.30 | **<0.001** | -5.68 | - | 0.036 |
| Language | 0.05 | 0.519 | 0.14 | 0.076 | -0.20 | **0.009** | 47.64 | - | 0.076 |
| Visuospatial | 0.14 | 0.068 | 0.06 | 0.488 | -0.06 | 0.422 | 61.64 | - | 0.488 |
| Executive | -0.02 | 0.739 | 0.03 | 0.738 | -0.28 | **<0.001** | 23.14 | - | 0.738 |

The results are based on general linear model for the rate/variability of cognitive changes. GLM 1 included a SAA positivity; GLM 2 included SAA positivity and right putaminal SRP; GLM 3 included SAA positivity and AD signature metabolism; and GLM 4 included all three predictors. All models were adjusted for baseline cognitive performance, age, sex, and education. P values for model comparison were obtained from likelihood ratio tests comparing GLM 1 vs. GLM 2 (P^1 vs. 2^) and GLM 3 vs. GLM 4 (P^3 vs. 4^), respectively. Multiple comparisons were corrected using the false discovery rate (FDR) across 12 cognitive outcomes. The p-values reported are uncorrected, and those that remained significant after FDR correction are shown in bold. Abbreviations: AD, Alzheimer’s disease; ADAS, Alzheimer’s disease assessment scale; AIC, Akaike information criterion; B, standardized beta coefficient; FDR, false discovery rate; MMSE, mini-mental state examination; Rt PUT SRP, right putaminal metabolism assessed by subject residual profile method; SAA, seed amplification assays.

**Supplementary Table 5.** Effects of SAA positivity, right putamen SRP, and AD signature metabolism on the rate/variability of cognitive changes in dementia due to AD participants

| Predictors | SAA positivity | | Rt PUT SRP | | AD signature metabolism | |  |  |  |
| --- | --- | --- | --- | --- | --- | --- | --- | --- | --- |
| Outcomes | B | P | B | P | B | P | AIC | P^1 vs. 2^ | P^3 vs. 4^ |
| **GLM 1** |  |  |  |  |  |  |  |  |  |
| *Annual change* |  |  |  |  |  |  |  |  |  |
| MMSE | -0.13 | 0.074 | - | - | - | - | 884.96 | **<0.001** | - |
| ADAS | 0.15 | 0.038 | - | - | - | - | 1220.59 | **0.010** | - |
| Memory | -0.21 | 0.004 | - | - | - | - | 237.94 | 0.570 | - |
| Language | -0.19 | 0.011 | - | - | - | - | 339.83 | 0.285 | - |
| Visuospatial | -0.09 | 0.239 | - | - | - | - | 494.18 | 0.312 | - |
| Executive | -0.15 | 0.049 | - | - | - | - | 285.52 | 0.389 | - |
| *Variability* |  |  |  |  |  |  |  |  |  |
| MMSE | 0.10 | 0.199 | - | - | - | - | 690.96 | **<0.001** | - |
| ADAS | 0.07 | 0.306 | - | - | - | - | 981.28 | **0.001** | - |
| Memory | 0.10 | 0.166 | - | - | - | - | 23.91 | 0.042 | - |
| Language | 0.04 | 0.583 | - | - | - | - | 70.72 | 0.049 | - |
| Visuospatial | 0.01 | 0.852 | - | - | - | - | 125.39 | 0.027 | - |
| Executive | 0.07 | 0.383 | - | - | - | - | 14.32 | 0.162 | - |
| **GLM 2** |  |  |  |  |  |  |  |  |  |
| *Annual change* |  |  |  |  |  |  |  |  |  |
| MMSE | -0.08 | 0.269 | -0.30 | **<0.001** | - | - | 868.65 | **<0.001** | - |
| ADAS | 0.12 | 0.098 | 0.19 | **0.010** | - | - | 1215.68 | **0.010** | - |
| Memory | -0.20 | 0.007 | -0.04 | 0.570 | - | - | 239.61 | 0.570 | - |
| Language | -0.17 | 0.021 | -0.08 | 0.285 | - | - | 340.64 | 0.285 | - |
| Visuospatial | -0.07 | 0.337 | -0.08 | 0.312 | - | - | 495.11 | 0.312 | - |
| Executive | -0.14 | 0.066 | -0.07 | 0.389 | - | - | 286.75 | 0.389 | - |
| *Variability* |  |  |  |  |  |  |  |  |  |
| MMSE | 0.05 | 0.529 | 0.30 | **<0.001** | - | - | 676.57 | **<0.001** | - |
| ADAS | 0.03 | 0.642 | 0.25 | **0.001** | - | - | 971.14 | **0.001** | - |
| Memory | 0.07 | 0.316 | 0.15 | 0.042 | - | - | 21.61 | 0.042 | - |
| Language | 0.01 | 0.858 | 0.15 | 0.049 | - | - | 68.67 | 0.049 | - |
| Visuospatial | -0.02 | 0.803 | 0.17 | 0.027 | - | - | 122.26 | 0.027 | - |
| Executive | 0.05 | 0.496 | 0.12 | 0.162 | - | - | 14.27 | 0.162 | - |
| **GLM 3** |  |  |  |  |  |  |  |  |  |
| *Annual change* |  |  |  |  |  |  |  |  |  |
| MMSE | -0.10 | 0.170 | - | - | 0.32 | **<0.001** | 867.69 | - | 0.005 |
| ADAS | 0.12 | 0.084 | - | - | -0.26 | **<0.001** | 1209.90 | - | 0.158 |
| Memory | -0.18 | 0.013 | - | - | 0.23 | **0.003** | 230.67 | - | 0.564 |
| Language | -0.16 | 0.028 | - | - | 0.20 | **0.009** | 334.66 | - | 0.913 |
| Visuospatial | -0.06 | 0.417 | - | - | 0.19 | **0.015** | 490.05 | - | 0.865 |
| Executive | -0.12 | 0.102 | - | - | 0.23 | **0.003** | 278.42 |  | 0.898 |
| *Variability* |  |  |  |  |  |  |  |  |  |
| MMSE | 0.05 | 0.444 | - | - | -0.40 | **<0.001** | 664.05 | - | 0.026 |
| ADAS | 0.03 | 0.687 | - | - | -0.44 | **<0.001** | 945.89 | - | 0.145 |
| Memory | 0.06 | 0.376 | - | - | -0.30 | **<0.001** | 9.61 | - | 0.512 |
| Language | 0.01 | 0.945 | - | - | -0.27 | **<0.001** | 59.83 | - | 0.455 |
| Visuospatial | -0.02 | 0.761 | - | - | -0.26 | **0.001** | 116.69 | - | 0.233 |
| Executive | 0.03 | 0.682 | - | - | -0.32 | **<0.001** | 0.51 | - | 0.896 |
| **GLM 4** |  |  |  |  |  |  |  |  |  |
| *Annual change* |  |  |  |  |  |  |  |  |  |
| MMSE | -0.07 | 0.323 | -0.21 | 0.005 | 0.23 | **0.003** | 861.36 | - | 0.005 |
| ADAS | 0.11 | 0.126 | 0.11 | 0.158 | -0.22 | **0.006** | 1209.81 | - | 0.158 |
| Memory | -0.19 | 0.011 | 0.05 | 0.564 | 0.24 | **0.003** | 232.32 | - | 0.564 |
| Language | -0.16 | 0.031 | -0.01 | 0.913 | 0.20 | **0.017** | 336.64 | - | 0.913 |
| Visuospatial | -0.06 | 0.439 | -0.01 | 0.865 | 0.19 | **0.028** | 492.02 | - | 0.865 |
| Executive | -0.12 | 0.102 | 0.01 | 0.898 | 0.24 | **0.005** | 280.41 | - | 0.898 |
| *Variability* |  |  |  |  |  |  |  |  |  |
| MMSE | 0.03 | 0.648 | 0.17 | 0.026 | -0.33 | **<0.001** | 660.83 | - | 0.026 |
| ADAS | 0.01 | 0.837 | 0.11 | 0.145 | -0.40 | **<0.001** | 945.66 | - | 0.145 |
| Memory | 0.06 | 0.435 | 0.05 | 0.512 | -0.28 | **0.001** | 11.16 | - | 0.512 |
| Language | -0.003 | 0.969 | 0.06 | 0.455 | -0.25 | **0.003** | 61.25 | - | 0.455 |
| Visuospatial | -0.04 | 0.631 | 0.10 | 0.233 | -0.22 | **0.010** | 117.21 | - | 0.233 |
| Executive | 0.03 | 0.694 | 0.01 | 0.896 | -0.31 | **<0.001** | 2.49 | - | 0.896 |

The results are based on general linear model for the rate/variability of cognitive changes. GLM 1 included a SAA positivity; GLM 2 included SAA positivity and right putaminal SRP; GLM 3 included SAA positivity and AD signature metabolism; and GLM 4 included all three predictors. All models were adjusted for baseline cognitive performance, age, sex, and education. P values for model comparison were obtained from likelihood ratio tests comparing GLM 1 vs. GLM 2 (P^1 vs. 2^) and GLM 3 vs. GLM 4 (P^3 vs. 4^), respectively. Multiple comparisons were corrected using the false discovery rate (FDR) across 12 cognitive outcomes. The p-values reported are uncorrected, and those that remained significant after FDR correction are shown in bold. Abbreviations: AD, Alzheimer’s disease; ADAS, Alzheimer’s disease assessment scale; AIC, Akaike information criterion; B, standardized beta coefficient; FDR, false discovery rate; MMSE, mini-mental state examination; Rt PUT SRP, right putaminal metabolism assessed by subject residual profile method; SAA, seed amplification assays.

**Supplementary Table 6.** Effects of SAA positivity, left middle occipital gyrus SUVR, and AD signature metabolism on the rate/variability of cognitive changes in MCI due to AD participants

| Predictors | SAA positivity | | Lt MOG SUVR | | AD signature metabolism | |  |  |  |
| --- | --- | --- | --- | --- | --- | --- | --- | --- | --- |
| Outcomes | B | P | B | P | B | P | AIC | P^1 vs. 2^ | P^3 vs. 4^ |
| **GLM 1** |  |  |  |  |  |  |  |  |  |
| *Annual change* |  |  |  |  |  |  |  |  |  |
| MMSE | -0.10 | 0.191 | - | - | - | - | 769.97 | **0.005** | - |
| ADAS | 0.20 | **0.008** | - | - | - | - | 1062.51 | **0.010** | - |
| Memory | -0.24 | **0.001** | - | - | - | - | 110.68 | **0.004** | - |
| Language | -0.11 | 0.137 | - | - | - | - | 246.09 | 0.035 | - |
| Visuospatial | -0.13 | 0.085 | - | - | - | - | 269.50 | 0.106 | - |
| Executive | -0.06 | 0.430 | - | - | - | - | 183.51 | **<0.001** | - |
| *Variability* |  |  |  |  |  |  |  |  |  |
| MMSE | 0.16 | 0.034 | - | - | - | - | 659.29 | 0.103 | - |
| ADAS | 0.12 | 0.108 | - | - | - | - | 953.63 | 0.097 | - |
| Memory | 0.06 | 0.438 | - | - | - | - | 12.98 | 0.069 | - |
| Language | 0.08 | 0.270 | - | - | - | - | 54.77 | 0.230 | - |
| Visuospatial | 0.15 | 0.040 | - | - | - | - | 58.98 | 0.773 | - |
| Executive | 0.01 | 0.910 | - | - | - | - | 32.64 | 0.055 | - |
| **GLM 2** |  |  |  |  |  |  |  |  |  |
| *Annual change* |  |  |  |  |  |  |  |  |  |
| MMSE | -0.06 | 0.405 | 0.21 | **0.005** | - | - | 763.93 | **0.005** | - |
| ADAS | 0.17 | 0.023 | -0.20 | **0.010** | - | - | 1057.55 | **0.010** | - |
| Memory | -0.21 | 0.005 | 0.21 | **0.004** | - | - | 104.10 | **0.004** | - |
| Language | -0.09 | 0.245 | 0.16 | 0.035 | - | - | 243.47 | 0.035 | - |
| Visuospatial | -0.11 | 0.145 | 0.12 | 0.106 | - | - | 268.78 | 0.106 | - |
| Executive | -0.02 | 0.824 | 0.32 | **<0.001** | - | - | 168.97 | **<0.001** | - |
| *Variability* |  |  |  |  |  |  |  |  |  |
| MMSE | 0.14 | 0.074 | -0.13 | 0.103 | - | - | 658.51 | 0.103 | - |
| ADAS | 0.10 | 0.180 | -0.13 | 0.097 | - | - | 952.76 | 0.097 | - |
| Memory | 0.04 | 0.640 | -0.14 | 0.069 | - | - | 11.53 | 0.069 | - |
| Language | 0.07 | 0.364 | -0.09 | 0.230 | - | - | 55.27 | 0.230 | - |
| Visuospatial | 0.15 | 0.048 | -0.02 | 0.773 | - | - | 60.89 | 0.773 | - |
| Executive | -0.01 | 0.872 | -0.16 | 0.055 | - | - | 30.81 | 0.055 | - |
| **GLM 3** |  |  |  |  |  |  |  |  |  |
| *Annual change* |  |  |  |  |  |  |  |  |  |
| MMSE | -0.06 | 0.399 | - | - | 0.26 | **<0.001** | 758.96 | - | 0.839 |
| ADAS | 0.17 | 0.019 | - | - | -0.27 | **<0.001** | 1051.36 | - | 0.598 |
| Memory | -0.21 | **0.004** | - | - | 0.29 | **<0.001** | 96.28 | - | 0.529 |
| Language | -0.09 | 0.214 | - | - | 0.19 | **0.017** | 242.14 | - | 0.787 |
| Visuospatial | -0.10 | 0.155 | - | - | 0.17 | **0.020** | 265.90 | - | 0.600 |
| Executive | -0.02 | 0.738 | - | - | 0.33 | **<0.001** | 167.59 |  | 0.255 |
| *Variability* |  |  |  |  |  |  |  |  |  |
| MMSE | 0.12 | 0.103 | - | - | -0.27 | **<0.001** | 647.30 | - | 0.016 |
| ADAS | 0.09 | 0.224 | - | - | -0.31 | **<0.001** | 938.92 | - | **0.004** |
| Memory | 0.02 | 0.785 | - | - | -0.32 | **<0.001** | -3.09 | - | **0.005** |
| Language | 0.06 | 0.412 | - | - | -0.21 | **0.006** | 48.93 | - | 0.068 |
| Visuospatial | 0.14 | 0.056 | - | - | -0.07 | 0.371 | 60.15 | - | 0.424 |
| Executive | -0.02 | 0.771 | - | - | -0.28 | **<0.001** | 21.26 | - | 0.115 |
| **GLM 4** |  |  |  |  |  |  |  |  |  |
| *Annual change* |  |  |  |  |  |  |  |  |  |
| MMSE | -0.06 | 0.391 | -0.03 | 0.839 | 0.28 | **0.029** | 760.91 | - | 0.839 |
| ADAS | 0.18 | 0.017 | 0.07 | 0.598 | -0.33 | **0.013** | 1053.07 | - | 0.598 |
| Memory | -0.21 | **0.003** | -0.08 | 0.529 | 0.35 | **0.005** | 97.86 | - | 0.529 |
| Language | -0.09 | 0.230 | 0.04 | 0.787 | 0.16 | 0.248 | 244.06 | - | 0.787 |
| Visuospatial | -0.11 | 0.145 | -0.07 | 0.600 | 0.23 | 0.082 | 267.62 | - | 0.600 |
| Executive | -0.02 | 0.818 | 0.15 | 0.255 | 0.21 | 0.106 | 168.23 | - | 0.255 |
| *Variability* |  |  |  |  |  |  |  |  |  |
| MMSE | 0.14 | 0.055 | 0.32 | 0.016 | -0.53 | **<0.001** | 643.16 | - | 0.016 |
| ADAS | 0.11 | 0.120 | 0.36 | **0.004** | -0.60 | **<0.001** | 932.46 | - | **0.004** |
| Memory | 0.04 | 0.554 | 0.36 | **0.005** | -0.61 | **<0.001** | -9.40 | - | **0.005** |
| Language | 0.08 | 0.303 | 0.24 | 0.068 | -0.40 | **0.002** | 47.44 | - | 0.068 |
| Visuospatial | 0.15 | 0.048 | 0.11 | 0.424 | -0.15 | 0.245 | 61.48 | - | 0.424 |
| Executive | -0.01 | 0.879 | 0.21 | 0.115 | -0.44 | **0.001** | 20.65 | - | 0.115 |

The results are based on general linear model for the rate/variability of cognitive changes. GLM 1 included a SAA positivity; GLM 2 included SAA positivity and left middle occipital gyrus SUVR; GLM 3 included SAA positivity and AD signature metabolism; and GLM 4 included all three predictors. All models were adjusted for baseline cognitive performance, age, sex, and education. P values for model comparison were obtained from likelihood ratio tests comparing GLM 1 vs. GLM 2 (P^1 vs. 2^) and GLM 3 vs. GLM 4 (P^3 vs. 4^), respectively. Multiple comparisons were corrected using the false discovery rate (FDR) across 12 cognitive outcomes. The p-values reported are uncorrected, and those that remained significant after FDR correction are shown in bold. Abbreviations: AD, Alzheimer’s disease; ADAS, Alzheimer’s disease assessment scale; AIC, Akaike information criterion; B, standardized beta coefficient; FDR, false discovery rate; MMSE, mini-mental state examination; Rt PUT SRP, right putaminal metabolism assessed by subject residual profile method; SAA, seed amplification assays.

**Supplementary Table 7.** Effects of SAA positivity, left middle occipital gyrus SUVR, and AD signature metabolism on the rate/variability of cognitive changes in dementia due to AD participants

| Predictors | SAA positivity | | Lt MOG SUVR | | AD signature metabolism | |  |  |  |
| --- | --- | --- | --- | --- | --- | --- | --- | --- | --- |
| Outcomes | B | P | B | P | B | P | AIC | P^1 vs. 2^ | P^3 vs. 4^ |
| **GLM 1** |  |  |  |  |  |  |  |  |  |
| *Annual change* |  |  |  |  |  |  |  |  |  |
| MMSE | -0.13 | 0.074 | - | - | - | - | 884.96 | 0.031 | - |
| ADAS | 0.15 | 0.038 | - | - | - | - | 1220.59 | 0.068 | - |
| Memory | -0.21 | 0.004 | - | - | - | - | 237.94 | 0.087 | - |
| Language | -0.19 | 0.011 | - | - | - | - | 339.83 | 0.214 | - |
| Visuospatial | -0.09 | 0.239 | - | - | - | - | 494.18 | 0.460 | - |
| Executive | -0.15 | 0.049 | - | - | - | - | 285.52 | 0.097 | - |
| *Variability* |  |  |  |  |  |  |  |  |  |
| MMSE | 0.10 | 0.199 | - | - | - | - | 690.96 | **<0.001** | - |
| ADAS | 0.07 | 0.306 | - | - | - | - | 981.28 | **<0.001** | - |
| Memory | 0.10 | 0.166 | - | - | - | - | 23.91 | **0.002** | - |
| Language | 0.04 | 0.583 | - | - | - | - | 70.72 | **0.002** | - |
| Visuospatial | 0.01 | 0.852 | - | - | - | - | 125.39 | **0.001** | - |
| Executive | 0.07 | 0.383 | - | - | - | - | 14.32 | **<0.001** | - |
| **GLM 2** |  |  |  |  |  |  |  |  |  |
| *Annual change* |  |  |  |  |  |  |  |  |  |
| MMSE | -0.10 | 0.153 | 0.16 | 0.031 | - | - | 882.08 | 0.031 | - |
| ADAS | 0.13 | 0.069 | -0.13 | 0.068 | - | - | 1219.11 | 0.068 | - |
| Memory | -0.19 | 0.011 | 0.13 | 0.087 | - | - | 236.90 | 0.087 | - |
| Language | -0.17 | 0.021 | 0.09 | 0.214 | - | - | 340.22 | 0.214 | - |
| Visuospatial | -0.08 | 0.301 | 0.06 | 0.460 | - | - | 495.61 | 0.460 | - |
| Executive | -0.13 | 0.074 | 0.13 | 0.097 | - | - | 284.65 | 0.097 | - |
| *Variability* |  |  |  |  |  |  |  |  |  |
| MMSE | 0.04 | 0.541 | -0.32 | **<0.001** | - | - | 673.73 | **<0.001** | - |
| ADAS | 0.03 | 0.636 | -0.30 | **<0.001** | - | - | 964.98 | **<0.001** | - |
| Memory | 0.06 | 0.377 | -0.23 | **0.002** | - | - | 15.45 | **0.002** | - |
| Language | 0.00 | 0.968 | -0.24 | **0.002** | - | - | 62.56 | **0.002** | - |
| Visuospatial | -0.03 | 0.679 | -0.26 | **0.001** | - | - | 114.88 | **0.001** | - |
| Executive | 0.03 | 0.638 | -0.30 | **<0.001** | - | - | 0.75 | **<0.001** | - |
| **GLM 3** |  |  |  |  |  |  |  |  |  |
| *Annual change* |  |  |  |  |  |  |  |  |  |
| MMSE | -0.10 | 0.170 | - | - | 0.32 | **<0.001** | 867.69 | - | 0.544 |
| ADAS | 0.12 | 0.084 | - | - | -0.26 | **<0.001** | 1209.90 | - | 0.721 |
| Memory | -0.18 | 0.013 | - | - | 0.23 | **0.003** | 230.67 | - | 0.883 |
| Language | -0.16 | 0.028 | - | - | 0.20 | **0.009** | 334.66 | - | 0.698 |
| Visuospatial | -0.06 | 0.417 | - | - | 0.19 | **0.015** | 490.05 | - | 0.399 |
| Executive | -0.12 | 0.102 | - | - | 0.23 | **0.003** | 278.42 |  | 0.823 |
| *Variability* |  |  |  |  |  |  |  |  |  |
| MMSE | 0.05 | 0.444 | - | - | -0.40 | **<0.001** | 664.05 | - | 0.152 |
| ADAS | 0.03 | 0.687 | - | - | -0.44 | **<0.001** | 945.89 | - | 0.392 |
| Memory | 0.06 | 0.376 | - | - | -0.30 | **<0.001** | 9.61 | - | 0.328 |
| Language | 0.01 | 0.945 | - | - | -0.27 | **<0.001** | 59.83 | - | 0.187 |
| Visuospatial | -0.02 | 0.761 | - | - | -0.26 | **0.001** | 116.69 | - | 0.048 |
| Executive | 0.03 | 0.682 | - | - | -0.32 | **<0.001** | 0.51 | - | 0.061 |
| **GLM 4** |  |  |  |  |  |  |  |  |  |
| *Annual change* |  |  |  |  |  |  |  |  |  |
| MMSE | -0.10 | 0.152 | -0.05 | 0.544 | 0.35 | **<0.001** | 869.31 | - | 0.544 |
| ADAS | 0.12 | 0.081 | 0.03 | 0.721 | -0.28 | **0.003** | 1211.77 | - | 0.721 |
| Memory | -0.18 | 0.013 | -0.01 | 0.883 | 0.24 | **0.015** | 232.65 | - | 0.883 |
| Language | -0.16 | 0.026 | -0.04 | 0.698 | 0.22 | **0.019** | 336.50 | - | 0.698 |
| Visuospatial | -0.07 | 0.372 | -0.08 | 0.399 | 0.24 | **0.014** | 491.31 | - | 0.399 |
| Executive | -0.12 | 0.101 | -0.02 | 0.823 | 0.25 | **0.015** | 280.37 | - | 0.823 |
| *Variability* |  |  |  |  |  |  |  |  |  |
| MMSE | 0.04 | 0.556 | -0.13 | 0.152 | -0.32 | **0.001** | 663.90 | - | 0.152 |
| ADAS | 0.02 | 0.743 | -0.07 | 0.392 | -0.39 | **<0.001** | 947.12 | - | 0.392 |
| Memory | 0.06 | 0.434 | -0.09 | 0.328 | -0.24 | **0.011** | 10.61 | - | 0.328 |
| Language | 0.00 | 0.948 | -0.12 | 0.187 | -0.20 | **0.037** | 60.01 | - | 0.187 |
| Visuospatial | -0.04 | 0.612 | -0.18 | 0.048 | -0.14 | 0.139 | 114.59 | - | 0.048 |
| Executive | 0.02 | 0.738 | -0.18 | 0.061 | -0.20 | 0.053 | -1.18 | - | 0.061 |

The results are based on general linear model for the rate/variability of cognitive changes. GLM 1 included a SAA positivity; GLM 2 included SAA positivity and left middle occipital gyrus SUVR; GLM 3 included SAA positivity and AD signature metabolism; and GLM 4 included all three predictors. All models were adjusted for baseline cognitive performance, age, sex, and education. P values for model comparison were obtained from likelihood ratio tests comparing GLM 1 vs. GLM 2 (P^1 vs. 2^) and GLM 3 vs. GLM 4 (P^3 vs. 4^), respectively. Multiple comparisons were corrected using the false discovery rate (FDR) across 12 cognitive outcomes. The p-values reported are uncorrected, and those that remained significant after FDR correction are shown in bold. Abbreviations: AD, Alzheimer’s disease; ADAS, Alzheimer’s disease assessment scale; AIC, Akaike information criterion; B, standardized beta coefficient; FDR, false discovery rate; MMSE, mini-mental state examination; Rt PUT SRP, right putaminal metabolism assessed by subject residual profile method; SAA, seed amplification assays.

**Supplementary Table 8.** Effect of SAA-positivity and amyloid PET centiloids on right putamen SRP or left middle occipital gyrus SUVR

|  | SAA positivity | | Amyloid CL | | Interaction | |
| --- | --- | --- | --- | --- | --- | --- |
|  | B | P | B | P | B | P |
| **Whole participants** |  |  |  |  |  |  |
| Rt PUT SRP | 0.14 | **0.004** | 0.09 | 0.054 | - | - |
| Lt MOG SUVR | −0.17 | **<0.001** | −0.08 | 0.097 | - | - |
| Lt MOG SUVR | −0.43 | **<0.001** | −0.15 | **0.006** | 0.31 | **0.005** |
| **AD participants** |  |  |  |  |  |  |
| Rt PUT SRP | 0.14 | **0.010** | 0.04 | 0.477 | - | - |
| Lt MOG SUVR | −0.17 | **0.002** | −0.03 | 0.525 | - | - |
| Lt MOG SUVR | −0.48 | **<0.001** | −0.13 | **0.038** | 0.36 | **0.009** |

The results are based on general linear models adjusted for age, sex, education, and baseline MMSE score. SAA-positivity and amyloid centiloids (CL) were included as predictors. Interaction terms between SAA-positivity and amyloid CL were included in the model when statistically significant. Abbreviations: AD, Alzheimer’s disease; B, standardized beta coefficient; CL, centiloids; Lt MOG SUVR, left middle occipital gyrus metabolism assessed by standardized uptake value ratio; Rt PUT SRP, right putaminal metabolism assessed by single residual profile method; SAA, seed amplification assays.

**Supplementary Table 9**. Effect of right putamen SRP and left middle occipital gyrus SUVR on longitudinal cognitive decline

|  | NC | | | | AD^SAA−^ | | | | AD^SAA+^ | | | |
| --- | --- | --- | --- | --- | --- | --- | --- | --- | --- | --- | --- | --- |
|  | Rt PUT SRP × Time | | Lt MOG SUVR × Time | | Rt PUT SRP × Time | | Lt MOG SUVR × Time | | Rt PUT SRP × Time | | Lt MOG SUVR × Time | |
|  | B (SE) | P | B (SE) | P | B (SE) | P | B (SE) | P | B (SE) | P | B (SE) | P |
| **Univariable model** |  |  |  |  |  |  |  |  |  |  |  |  |
| MMSE | -0.22 (0.46) | 0.626 | 0.24 (0.25) | 0.350 | -7.10 (1.59) | **<0.001** | 4.70 (0.76) | **<0.001** | -10.74 (2.83) | **<0.001** | 4.22 (1.82) | **0.024** |
| ADAS | 0.78 (1.01) | 0.444 | 0.50 (0.56) | 0.374 | 12.74 (3.23) | **<0.001** | -8.28 (1.57) | **<0.001** | 18.58 (5.05) | **0.001** | -7.61 (3.18) | **0.021** |
| Memory | -0.28 (0.17) | 0.109 | -0.10 (0.10) | 0.314 | -0.82 (0.24) | **0.001** | 0.62 (0.12) | **<0.001** | -0.51 (0.35) | 0.145 | 0.27 (0.21) | 0.209 |
| Language | -0.22 (0.16) | 0.173 | -0.07 (0.09) | 0.459 | -0.97 (0.29) | **0.001** | 0.75 (0.14) | **<0.001** | -1.21 (0.48) | **0.014** | 0.45 (0.30) | 0.143 |
| Visuospatial | -0.10 (0.18) | 0.569 | -0.06 (0.10) | 0.541 | -0.54 (0.27) | 0.049 | 0.45 (0.13) | **0.001** | -0.52 (0.47) | 0.280 | 0.62 (0.27) | **0.029** |
| Executive | -0.29 (0.14) | 0.044 | 0.07 (0.08) | 0.416 | -0.34 (0.27) | 0.218 | 0.68 (0.13) | **<0.001** | -0.65 (0.44) | 0.145 | 0.66 (0.26) | **0.014** |
| **Multivariable model** |  |  |  |  |  |  |  |  |  |  |  |  |
| MMSE | -0.22 (0.46) | 0.641 | 0.24 (0.26) | 0.345 | -4.56 (1.56) | **0.004** | 4.03 (0.79) | **<0.001** | -9.53 (3.16) | **0.004** | 1.64 (1.95) | 0.405 |
| ADAS | 0.76 (1.02) | 0.456 | 0.49 (0.57) | 0.387 | 8.36 (3.23) | **0.011** | -6.95 (1.63) | **<0.001** | 15.70 (5.58) | **0.007** | -3.47 (3.46) | 0.321 |
| Memory | -0.28 (0.17) | 0.117 | -0.09 (0.10) | 0.329 | -0.48 (0.25) | 0.055 | 0.54 (0.12) | **<0.001** | -0.36 (0.39) | 0.354 | 0.17 (0.24) | 0.474 |
| Language | -0.22 (0.16) | 0.180 | -0.06 (0.09) | 0.479 | -0.55 (0.28) | 0.053 | 0.66 (0.14) | **<0.001** | -1.09 (0.54) | 0.045 | 0.17 (0.33) | 0.619 |
| Visuospatial | -0.07 (0.21) | 0.731 | -0.06 (0.12) | 0.640 | -0.27 (0.27) | 0.324 | 0.41 (0.14) | **0.004** | 0.02 (0.69) | 0.976 | 0.68 (0.43) | 0.119 |
| Executive | -0.30 (0.14) | 0.045 | 0.07 (0.08) | 0.395 | 0.11 (0.27) | 0.694 | 0.70 (0.13) | **<0.001** | -0.20 (0.48) | 0.672 | 0.63(0.29) | 0.035 |

Linear mixed models were used to assess the effects of regional brain metabolism on longitudinal cognitive trajectories. Predictors included Time × right putamen SRP and Time × left middle occipital gyrus SUVR interaction terms. Univariable models included one region and its interaction with time, while multivariable models included both regions simultaneously. All models adjusted for baseline cognitive performance, age, sex, and education, with participant-level random intercepts and slopes for time. Multiple comparisons were corrected using the false discovery rate (FDR) across 6 cognitive outcomes each group. The p values reported are uncorrected, and those that remained significant after FDR correction are shown in bold. Abbreviations: AD, Alzheimer’s disease; ADAS, Alzheimer’s disease assessment scale; B, standardized beta coefficient; FDR, false discovery rate; Lt MOG SUVR, left middle occipital gyrus metabolism assessed by standardized uptake value ratio; MMSE, mini-mental state examination; NC, normal control; PTAR, phosphorylated tau/Aβ42 ratio; Rt PUT SRP, right putaminal metabolism assessed by subject residual profile method; SAA, seed amplification assays.

**Supplementary Table 10.** Association between right putamen SRP or left middle occipital gyrus SUVR and cognitive variability

|  | NC | | | | AD^SAA−^ | | | | AD^SAA+^ | | | |
| --- | --- | --- | --- | --- | --- | --- | --- | --- | --- | --- | --- | --- |
|  | Rt PUT SRP | | Lt MOG SUVR | | Rt PUT SRP | | Lt MOG SUVR | | Rt PUT SRP | | Lt MOG SUVR | |
|  | B | P | B | P | B | P | B | P | B | P | B | P |
| **Univariable model** |  |  |  |  |  |  |  |  |  |  |  |  |
| MMSE | 0.13 | 0.262 | 0.20 | 0.036 | 0.16 | 0.020 | -0.27 | **<0.001** | 0.27 | **0.006** | -0.21 | **0.025** |
| ADAS | 0.06 | 0.581 | -0.07 | 0.473 | 0.21 | **0.002** | -0.17 | **0.010** | 0.30 | **0.002** | -0.35 | **<0.001** |
| Memory | 0.15 | 0.181 | -0.11 | 0.221 | 0.14 | 0.041 | -0.17 | **0.009** | 0.13 | 0.201 | -0.09 | 0.320 |
| Language | 0.11 | 0.335 | -0.01 | 0.918 | 0.07 | 0.288 | -0.15 | **0.025** | 0.24 | **0.011** | -0.13 | 0.163 |
| Visuospatial | 0.12 | 0.244 | 0.15 | 0.099 | 0.13 | 0.050 | -0.14 | **0.034** | 0.16 | 0.122 | -0.25 | **0.010** |
| Executive | 0.15 | 0.189 | 0.08 | 0.418 | 0.04 | 0.556 | -0.26 | **<0.001** | 0.11 | 0.294 | -0.10 | 0.303 |
| **Multivariable model** |  |  |  |  |  |  |  |  |  |  |  |  |
| MMSE | 0.14 | 0.228 | 0.21 | 0.032 | 0.10 | 0.143 | -0.25 | **<0.001** | 0.21 | 0.042 | -0.13 | 0.189 |
| ADAS | 0.06 | 0.619 | -0.06 | 0.499 | 0.18 | **0.007** | -0.13 | 0.044 | 0.18 | 0.064 | -0.29 | **0.002** |
| Memory | 0.14 | 0.204 | -0.11 | 0.250 | 0.11 | 0.123 | -0.15 | **0.024** | 0.10 | 0.337 | -0.05 | 0.594 |
| Language | 0.11 | 0.339 | -0.01 | 0.959 | 0.04 | 0.538 | -0.14 | 0.040 | 0.23 | 0.030 | -0.05 | 0.636 |
| Visuospatial | 0.13 | 0.205 | 0.15 | 0.085 | 0.10 | 0.142 | -0.11 | 0.093 | 0.07 | 0.492 | -0.22 | 0.030 |
| Executive | 0.16 | 0.174 | 0.09 | 0.376 | -0.01 | 0.851 | -0.26 | **<0.001** | 0.08 | 0.455 | -0.07 | 0.472 |

The results are based on general linear models using right putamen SRP or left middle occipital gyrus SUVR as predictors.

The univariable model included either right putamen SRP or left middle occipital gyrus SUVR as a predictor, whereas the multivariable model includes both as predictors. Covariates include age, sex, education, and baseline cognitive scores (e.g., baseline MMSE, baseline ADAS). Multiple comparisons were corrected using the FDR method across 6 cognitive outcomes within each group. The p values reported are uncorrected, and those that remained significant after FDR correction are shown in bold. Abbreviations: AD, Alzheimer’s disease; ADAS, Alzheimer’s disease assessment scale; B, standardized beta coefficient; FDR, false discovery rate; Lt MOG SUVR, left middle occipital gyrus metabolism assessed by standardized uptake value ratio; MMSE, mini-mental state examination; NC, normal control; Rt PUT SRP, right putaminal metabolism assessed by subject residual profile method; SAA, seed amplification assays.

**Supplementary Table 11**. Effects of SAA positivity, right putamen SRP, and AD signature metabolism on longitudinal cognitive decline in AD participants

| Predictors | SAA positivity × Time | | Rt PUT SRP × Time | | AD signature metabolism × Time | |  |  |  |
| --- | --- | --- | --- | --- | --- | --- | --- | --- | --- |
| Outcomes | Beta (SE) | P | Beta (SE) | P | Beta (SE) | P | AIC | P^1 vs. 2^ | P^3 vs. 4^ |
| **LLM 1** |  |  |  |  |  |  |  |  |  |
| MMSE | -0.82 (0.24) | **0.001** | - | - | - | - | 8061.22 | **<0.001** | - |
| ADAS | 1.46 (0.46) | **0.002** | - | - | - | - | 10311.11 | **<0.001** | - |
| Memory | -0.10 (0.03) | **0.003** | - | - | - | - | 1715.70 | **0.001** | - |
| Language | -0.12 (0.04) | **0.004** | - | - | - | - | 2280.81 | **<0.001** | - |
| Visuospatial | -0.10 (0.04) | **0.014** | - | - | - | - | 3517.11 | **0.003** | - |
| Executive | -0.05 (0.04) | 0.183 | - | - | - | - | 2179.69 | **0.008** | - |
| **LLM 2** |  |  |  |  |  |  |  |  |  |
| MMSE | -0.54 (0.23) | 0.019 | -8.29 (1.40) | **<0.001** | - | - | 8023.62 | **<0.001** | - |
| ADAS | 0.97 (0.45) | 0.032 | 14.79 (2.71) | **<0.001** | - | - | 10278.92 | **<0.001** | - |
| Memory | -0.08 (0.03) | 0.020 | -0.72 (0.20) | **<0.001** | - | - | 1708.02 | **0.001** | - |
| Language | -0.09 (0.04) | 0.037 | -1.05 (0.25) | **<0.001** | - | - | 2266.37 | **<0.001** | - |
| Visuospatial | -0.08 (0.04) | 0.045 | -0.55 (0.23) | **0.019** | - | - | 3511.16 | **0.003** | - |
| Executive | -0.04 (0.04) | 0.337 | -0.45 (0.23) | 0.054 | - | - | 2176.37 | **0.008** | - |
| **LLM 3** |  |  |  |  |  |  |  |  |  |
| MMSE | -0.42 (0.21) | 0.045 | - | - | 6.70 (0.67) | **<0.001** | 7971.24 | - | **0.003** |
| ADAS | 0.74 (0.40) | 0.070 | - | - | -12.51 (1.31) | **<0.001** | 10231.07 | - | 0.025 |
| Memory | -0.05 (0.03) | 0.073 | - | - | 0.80 (0.10) | **<0.001** | 1665.39 | - | 0.253 |
| Language | -0.06 (0.04) | 0.088 | - | - | 1.01 (0.12) | **<0.001** | 2221.42 | - | 0.089 |
| Visuospatial | -0.06 (0.04) | 0.107 | - | - | 0.62 (0.12) | **<0.001** | 3477.75 | - | 0.385 |
| Executive | -0.004 (0.03) | 0.908 | - | - | 0.87 (0.11) | **<0.001** | 2123.80 | - | 0.144 |
| **LLM 4** |  |  |  |  |  |  |  |  |  |
| MMSE | -0.33 (0.21) | 0.116 | -4.09 (1.33) | **0.002** | 5.92 (0.71) | **<0.001** | 7959.23 | - | **0.003** |
| ADAS | 0.59 (0.41) | 0.144 | 6.74 (2.59) | **0.010** | -11.18 (1.4) | **<0.001** | 10220.51 | - | 0.025 |
| Memory | -0.05 (0.03) | 0.102 | -0.20 (0.20) | 0.308 | 0.75 (0.1) | **<0.001** | 1669.50 | - | 0.253 |
| Language | -0.06 (0.04) | 0.143 | -0.41 (0.24) | 0.090 | 0.93 (0.13) | **<0.001** | 2222.80 | - | 0.089 |
| Visuospatial | -0.06 (0.04) | 0.129 | -0.14 (0.24) | 0.561 | 0.59 (0.13) | **<0.001** | 3481.38 | - | 0.385 |
| Executive | -0.01 (0.04) | 0.842 | 0.15 (0.22) | 0.490 | 0.90 (0.12) | **<0.001** | 2126.26 | - | 0.144 |

The results are based on linear mixed models evaluating longitudinal cognitive trajectories. LLM 1 included a Time × SAA positivity interaction; LLM 2 included Time × SAA positivity and Time × right putaminal SRP interactions; LLM 3 included Time × SAA positivity and Time × AD signature metabolism interactions; and LLM 4 included all three interaction terms. All models were adjusted for baseline cognitive performance, age, sex, and education, with participant-level random intercepts and random slopes for time. P values for model comparison were obtained from likelihood ratio tests comparing LLM 1 vs. LLM 2 (P^1 vs. 2^) and LLM 3 vs. LLM 4 (P^3 vs. 4^), respectively. Multiple comparisons were corrected using the false discovery rate (FDR) across 6 cognitive outcomes. The p-values reported are uncorrected, and those that remained significant after FDR correction are shown in bold. Abbreviations: AD, Alzheimer’s disease; ADAS, Alzheimer’s disease assessment scale; AIC, Akaike information criterion; B, standardized beta coefficient; FDR, false discovery rate; MMSE, mini-mental state examination; Rt PUT SRP, right putaminal metabolism assessed by subject residual profile method; SAA, seed amplification assays.

**Supplementary Table 12**. Effects of SAA positivity, left middle occipital gyrus SUVR, and AD signature metabolism on longitudinal cognitive decline in AD participants

| Predictors | SAA positivity × Time | | Lt MOG SUVR × Time | | AD signature metabolism × Time | |  |  |  |
| --- | --- | --- | --- | --- | --- | --- | --- | --- | --- |
| Outcomes | Beta (SE) | P | Beta (SE) | P | Beta (SE) | P | AIC | P^1 vs. 2^ | P^3 vs. 4^ |
| **LLM 1** |  |  |  |  |  |  |  |  |  |
| MMSE | -0.82 (0.24) | **0.001** | - | - | - | - | 8061.22 | **<0.001** | - |
| ADAS | 1.46 (0.46) | **0.002** | - | - | - | - | 10311.11 | **<0.001** | - |
| Memory | -0.10 (0.03) | **0.003** | - | - | - | - | 1715.70 | **<0.001** | - |
| Language | -0.12 (0.04) | **0.004** | - | - | - | - | 2280.81 | **<0.001** | - |
| Visuospatial | -0.10 (0.04) | **0.014** | - | - | - | - | 3517.11 | **<0.001** | - |
| Executive | -0.05 (0.04) | 0.183 | - | - | - | - | 2179.69 | **<0.001** | - |
| **LLM 2** |  |  |  |  |  |  |  |  |  |
| MMSE | -0.48 (0.23) | 0.037 | 4.54 (0.74) | **<0.001** | - | - | 8027.26 | **<0.001** | - |
| ADAS | 0.83 (0.45) | 0.065 | -8.19 (1.42) | **<0.001** | - | - | 10279.32 | **<0.001** | - |
| Memory | -0.06 (0.03) | 0.080 | 0.54 (0.1) | **<0.001** | - | - | 1699.28 | **<0.001** | - |
| Language | -0.07 (0.04) | 0.081 | 0.67 (0.13) | **<0.001** | - | - | 2260.62 | **<0.001** | - |
| Visuospatial | -0.05 (0.04) | 0.163 | 0.49 (0.12) | **<0.001** | - | - | 3492.12 | **<0.001** | - |
| Executive | -0.001 (0.04) | 0.985 | 0.68 (0.12) | **<0.001** | - | - | 2151.27 | **<0.001** | - |
| **LLM 3** |  |  |  |  |  |  |  |  |  |
| MMSE | -0.42 (0.21) | 0.045 | - | - | 6.70 (0.67) | **<0.001** | 7971.24 | - | 0.139 |
| ADAS | 0.74 (0.40) | 0.070 | - | - | -12.51 (1.31) | **<0.001** | 10231.07 | - | 0.324 |
| Memory | -0.05 (0.03) | 0.073 | - | - | 0.80 (0.10) | **<0.001** | 1665.39 | - | 0.445 |
| Language | -0.06 (0.04) | 0.088 | - | - | 1.01 (0.12) | **<0.001** | 2221.42 | - | 0.537 |
| Visuospatial | -0.06 (0.04) | 0.107 | - | - | 0.62 (0.12) | **<0.001** | 3477.75 | - | 0.412 |
| Executive | -0.004 (0.03) | 0.908 | - | - | 0.87 (0.11) | **<0.001** | 2123.80 | - | 0.904 |
| **LLM 4** |  |  |  |  |  |  |  |  |  |
| MMSE | -0.45 (0.21) | 0.035 | -1.22 (1.02) | 0.234 | 7.62 (1.02) | **<0.001** | 7968.209 | - | 0.139 |
| ADAS | 0.83 (0.41) | 0.046 | 2.84 (1.99) | 0.154 | -14.69 (2.01) | **<0.001** | 10226.816 | - | 0.324 |
| Memory | -0.06 (0.03) | 0.060 | -0.13 (0.15) | 0.368 | 0.9 (0.15) | **<0.001** | 1671.907 | - | 0.445 |
| Language | -0.07 (0.04) | 0.061 | -0.2 (0.18) | 0.284 | 1.16 (0.18) | **<0.001** | 2227.589 | - | 0.537 |
| Visuospatial | -0.06 (0.04) | 0.128 | 0.07 (0.17) | 0.681 | 0.56 (0.17) | **<0.001** | 3482.865 | - | 0.412 |
| Executive | -0.001 (0.04) | 0.968 | 0.07 (0.17) | 0.662 | 0.81 (0.17) | **<0.001** | 2131.135 | - | 0.904 |

The results are based on linear mixed models evaluating longitudinal cognitive trajectories. LLM 1 included a Time × SAA positivity interaction; LLM 2 included Time × SAA positivity and Time × left MOG SUVR interactions; LLM 3 included Time × SAA positivity and Time × AD signature metabolism interactions; and LLM 4 included all three interaction terms. All models were adjusted for baseline cognitive performance, age, sex, and education, with participant-level random intercepts and random slopes for time. P values for model comparison were obtained from likelihood ratio tests comparing LLM 1 vs. LLM 2 (P^1 vs. 2^) and LLM 3 vs. LLM 4 (P^3 vs. 4^), respectively. Multiple comparisons were corrected using the false discovery rate (FDR) across 6 cognitive outcomes. The p-values reported are uncorrected, and those that remained significant after FDR correction are shown in bold. Abbreviations: AD, Alzheimer’s disease; ADAS, Alzheimer’s disease assessment scale; AIC, Akaike information criterion; B, standardized beta coefficient; FDR, false discovery rate; Lt MOG SUVR, left middle occipital gyrus metabolism assessed by standardized uptake value ratio; MMSE, mini-mental state examination; SAA, seed amplification assays.
